# Individual and Comorbid Influences of Chronic Stress and a Western Diet on Allostatic Loads and Cardiac Resilience, Adaptation and Proteome Profiles in Male Mice

**DOI:** 10.1002/cph4.70045

**Published:** 2025-09-08

**Authors:** Makayla Nicholas, Tessa Helman, Brock Lyon, Saba Naghipour, Trissha Ybanez, Joshua T. Ingles, Chulkyu Kim, Nicolas J. C. Stapelberg, Jason N. Peart, John P. Headrick, Eugene F. du Toit

**Affiliations:** ^1^ School of Pharmacy and Medical Sciences Griffith University Southport Queensland Australia; ^2^ Centre for Healthy Brain Ageing (CHeBA), School of Clinical Medicine University of NSW Sydney New South Wales Australia; ^3^ O'Brien Institute Department St Vincent's Institute of Medical Research Fitzroy Victoria Australia; ^4^ Faculty of Health Sciences and Medicine Bond University Robina Queensland Australia

**Keywords:** adaptability, allostasis, anhedonia, cardiac ischemia, depression, diet, mitochondria, preconditioning, resilience, stress

## Abstract

Mechanisms underlying cardiovascular, affective, and metabolic (CAM) multimorbidity are incompletely defined. We assessed how two risk factors—chronic stress (CS) and a Western diet (WD)—interact to influence cardiovascular function, resilience, adaptability, and allostatic load (AL); explore pathway involvement; and examine relationships with behavioral, metabolic, and systemic AL. Male C57Bl/6 mice (8 weeks old, *n* = 64) consumed a control (CD) or WD (12%–65%–23% or 32%–57%–11% calories from fat‐carbohydrate‐protein) for 17 weeks, with half subjected to 2 h daily restraint stress over the final 2 weeks (CD + CS and WD + CS). The WD induced a pre‐diabetic state (increased weight, hyperinsulinemia, insulin‐resistance, and hyperleptinemia) and anhedonia, while CS reduced body weight and leptin levels and was anxiogenic. The cardiovascular system was particularly stress sensitive: the WD worsened resilience (vulnerability to ischemia–reperfusion; I‐R), while CS increased cardiac automaticity, reduced contractility and relaxation, worsened postischemic inflammation (TNF‐α) and inhibited adaptive resilience (efficacy of ischemic preconditioning; PC). Proteomics identified mitochondrial function, innate immunity, and xenobiotic metabolism as prominently modified processes. Regarding allostatic loading: comorbid CS and WD feeding were necessary to increase cardiovascular AL and additively increased behavioral AL; the WD (not CS) increased metabolic, neuroendocrine‐immune, and systemic AL. Summarizing: (i) distinct changes arise with CS (disrupted cardiac function, inflammation, adaptation; weight loss; anxiogenesis) versus a WD (reduced cardiac resilience; prediabetes; anhedonia); (ii) comorbid WD and CS additively worsen cardiovascular and behavioral AL; and (iii) mitochondrial and innate defense processes may underlie cardiac effects of diet and stress. Data support positive diet‐stress interactions that particularly increase cardiovascular and affective vulnerability.

## Graphical abstract

These findings support positive interactions between common risk factors in comorbid development of cardiovascular and affective disorders. Mitochondrial and innate defense paths may contribute to the influences of diet, stress, and preconditioning.

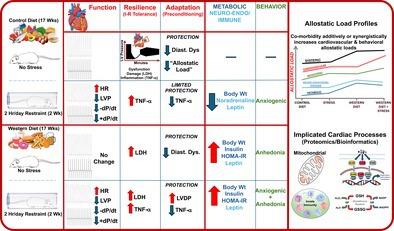

## 1 Introduction

Multimorbidity is the “new normal” for people with chronic disease (Tran et al. 2018). A significant proportion of patients over the age of 18–20 years experience multiple disorders (Ioakeim‐Skoufa et al. 2025; Kuan et al. 2023): ~70% of those with incident cardiovascular disease (CVD) have ≥ 2 comorbid conditions (Tran et al. 2018); individuals with major depressive disorder (MDD) are increasingly likely to suffer comorbid CVD and metabolic disorders; and these links have increased over recent decades (Dibato et al. 2021; Hare et al. 2014; Vancampfort et al. 2016). The growing prevalence of multimorbidity poses a major clinical challenge, escalating the complexity and cost of health care, and worsening patient outcomes. Individuals with multimorbidity face higher risks of hospitalization and more prolonged stays, as well as significantly higher morbidity and premature mortality (Menotti et al. 2001; Vogeli et al. 2007). Quality of life declines, coupled with a rise in MDD, polypharmacy, and socioeconomic costs (Fortin et al. 2004; Lehnert et al. 2011; Marengoni et al. 2011; Read et al. 2017; Ryan et al. 2015; Townsend et al. 2003). These impacts, and the increasing incidence of multimorbidity, demand a strategic shift in clinical practice—from a conventional single‐disease focus to patient‐centered models that address the growing complexity of individual profiles (Guthrie et al. 2012; Hughes et al. 2013; Smith et al. 2010). Moreover, primary preventative measures become increasingly vital in curtailing this profound yet largely preventable health challenge. Improved preventive and therapeutic strategies require an understanding of the mechanistic basis of CAM multimorbidity, and how common environmental stressors interact to drive this and other multimorbid conditions.

While the pathogenesis of CAM multimorbidity requires clarification, these prominent conditions share key features: (i) they are all complex multi‐system disorders, highlighting the integrative/systemic nature of our most burdensome diseases; (ii) this involves shared interorgan (e.g., Microbiome–gut–brain–cardiovascular) signaling axes (Patist et al. 2018; Rossi et al. 2022) and biological networks, as described for example in the psycho‐immuno‐neuroendocrine or chronic illness risk networks (which may be pluripotent for multiple disorders) (Stapelberg et al. 2019); and (iii) they are also associated with low‐grade inflammation, with inflammaging forwarded as an important linkage (Ajoolabady et al. 2024). More proximately, these disorders all share common lifestyle risks or environmental drivers, explaining much of their prevalence and substantial preventability. For example, 80% of coronary artery disease (Stampfer et al. 2000) and 90% of type 2 diabetes mellitus (Hu et al. 2001) may be attributable to modifiable environmental factors, while MDD is estimated to be ~60% preventable versus a highly polygenic risk (up to 300 gene‐variants or gene–environment interactions, with small odds ratios < 1.05) that may contribute to ≤ 40% heritability (Kendall et al. 2021; Major Depressive Disorder Working Group of the Psychiatric Genomics Consortium. Major Depressive Disorder Working Group of the Psychiatric Genomics Consortium 2025).

Shared environmental determinants and a high degree of preventability suggest a more fundamental link between CAM disorders, which may each stem from an evolutionary mismatch between our Paleolithic phenome and contemporary environments. This biological clash is exaggerated by select gene variants in secondary subsets of the population. Key (and shared) environmental determinants include a Western‐type diet, chronic psychosocial stress, socioeconomic disadvantage, pollution, smoking/drug use, and low physical activity. Indeed, Booth and colleagues convincingly prosecute the case that the latter is an important driver of many “diseases of modernity” (Booth et al. 2000; Booth and Lees 2007; Booth et al. 2012). Given the primacy of the environment‐phenome mismatch across CAM disorders, an effective strategy is to define how these interrelated environmental factors collectively drive multimorbidity, enabling/informing more targeted modification of external risk factors (and development of therapeutic interventions). However, relatively few studies address this critical question of how multiple environmental stressors interact in the pathogenesis of individual and multi‐morbid chronic diseases. This question may be most effectively interrogated within the integrative systems biology frameworks of allostasis (McEwen and Wingfield 2003; McEwen 1998), adaptive homeostasis (Davies 2016), or reactive scope (Romero et al. 2009).

Environmental stressors are thought to trigger allostatic (McEwen and Wingfield 2003; McEwen 1998) or adaptive homeostatic (Davies 2016) responses—shifts in homeostatic ranges that may improve survival in the short term, yet involve significant physiological trade‐offs. For example, allostatic responses may protect cerebral energy metabolism while predisposing to obesity and hyperglycemia (contributing to obesity and diabetes “paradoxes” (Peters and McEwen 2012; Tremblay and Chaput 2012)). These adaptations come with a cumulative biological cost, which may commence in early life (Mariani et al. 2021) and accrue as individuals experience repeated/chronic exposures to psychosocial, metabolic, and other stressors (Danese and McEwen 2012; McEwen and Wingfield 2003). This wear‐and‐tear ‐ “allostatic load” or “homeostatic overload”—may ultimately impair functionality, adaptation, and resilience to promote biological aging and chronic disease development (Danese and McEwen 2012; Davies 2016; Pomatto and Davies 2017). Our earlier work confirms a link between cardiac aging and declining resilience/adaptation, potentially involving shifts in energy metabolism, innate defenses, and quality control/repair processes (Ashton et al. 2023; Peart et al. 2014). However, interactions between common stressors in determining allostatic loads and multi‐morbid disease development remain unclear, and experimental findings may be contradictory. For example, some work suggests psychosocial and metabolic stressors interact synergistically (Du Toit et al. 2020; Fagundes and Wu 2021), reflecting shared mechanistic origins and neuroendocrine and metabolic sequelae (Patist et al. 2018; Stapelberg et al. 2019). Others document benign or even beneficial influences of stress on metabolic risk (Finger et al. 2012; Kai et al. 2000; Nemati et al. 2017). Early exposure to a high fat/sugar diet may induce stress tolerance (Kanazawa et al. 2003; Maniam et al. 2016), while stress may in turn induce resistance to metabolic dysfunction with such diets (Maniam et al. 2015). Our own work suggests CS may improve select metabolic risk factors in adult mice consuming an obesogenic diet (Hatton‐Jones et al. 2022), whereas early life stress exacerbates the behavioral and metabolic consequences of WD feeding (Robertson et al. 2024). Differences in outcomes may thus stem in part from the timing and nature of stress exposure, with early life having a lasting impact on the developing brain, while chronic stress in adulthood may interact with adaptive systems to somewhat mitigate negative impacts on metabolic health.

Given these uncertainties and conflicting observations, and the urgent need to better understand multimorbidity, we test whether two of the most widely shared environmental stressors—a Western‐type diet and psychosocial stress—interact in an additive or synergistic manner to drive cardiac dysfunction, allostatic loading, and CAM multimorbidity. We address three interrelated questions: (i) do chronic psychosocial and metabolic or dietary stressors interact to disrupt system functionality, resilience, and adaptability (as predicted in models of allostasis/adaptive homeostasis/reactive scope, and evident with advancing age); (ii) what molecular pathways may be involved (models predict disruption of energy generation and processes governing resilience/adaptation); and (iii) at an integrated systems level, how do psychosocial and dietary stress interact to influence cardiovascular, together with behavioral, metabolic, neuro‐endocrine‐immune, and overall systemic AL (models predict multi‐system disturbances favoring multi‐morbid disease development). We focus on the heart as a model system for tackling the first and second questions, while assessing relevant subsystem (cardiovascular, metabolic, neuro‐endocrine‐immune, and behavioral) and systemic responses in addressing the third.

## 2 Methods

### 2.1 Animal Ethics

Studies were approved by and performed in accordance with the guidelines of the Animal Ethics Committee of Griffith University (MSC/02/20/AEC), accredited by the Queensland Government, Department of Primary Industries and Fisheries under the guidelines of “The Animal Care and Protection Act 2001, Section 757.”

### 2.2 Experimental Design

A total of 64 8‐week old male C57Bl/6J mice were purchased from the Animal Resources Centre (ARC, Perth, Western Australia). Mice were randomly allocated into four experimental groups (4 mice/cage): (1) Control diet (CD); (2) WD; (3) CD + Chronic stress (CS—2‐h daily restraint per day for 14 days); and (4) WD + CS. Green Line GM500 cages separately ventilated in DGM racks (Techniplast S.p.A, Varese, Italy) were used to house mice, with ad libitum access to food and water (and daily standard care). Housing conditions included a 12‐h day–night cycle (lights on at 0700 h), 21°C ± 2°C temperature, and 40% ± 2% humidity. Mice were weighed daily for 12 weeks, then weekly after that. Daily weighing resumed during stress induction for mice in the CD + CS and WD + CS groups, and the post‐intervention behavioral analysis week for CD and WD mice.

### 2.3 Control and WD Feeding

The CD mice were fed a standard chow diet (Irradiated Rat and Mouse Cubes, Specialty Feeds, Glen Forrest, Western Australia) with 12%, 65%, and 23% of caloric content derived from fats, carbohydrates, and protein, respectively. The WD mice were fed a modified chow (formulation detailed below) with 32%, 57%, and 11% of calories from fats, carbohydrates, and protein, respectively. Each cage of WD mice was provided 40–50 g of fresh food daily (placed in cages between 1030 and 1230 h), and consumption was recorded each day. Mice were fed the WD for 17 weeks to induce a pre or early type‐2 diabetic state. Formulation of WD chow is detailed in Data S1.

The WD feed was formulated from: 250 g of solidified edible animal fat and oil (Supafry, Goodman Fielder Consumer Foods, Sydney, Australia); 280 g refined sugar (Coles Group, Victoria, Australia); 1588 g of sweetened condensed milk (Coles Group, Victoria, Australia); and 1600 g of powdered standard laboratory chow (control diet) (Irradiated Rat and Mouse Cubes, Specialty Feeds, Glen Forrest, Western Australia). Mixed to a dough‐like consistency, the food was stored at 4°C until use. We have previously employed a similar diet to promote obesity and a prediabetic phenotype (Du Toit et al. 2020; Hatton‐Jones et al. 2022; Robertson et al. 2024).

### 2.4 Chronic Mild Stress

The CS protocol was introduced over the final 14 days of the experimental period. The stress protocol involved restraining mice within a ventilated clear Perspex restraint device (3 cm diameter × 10 cm length) for 2 h daily (between 0900 and 1100 h). Restraint devices were placed in individual ventilated cages during the protocol.

### 2.5 Assessment of Behavioral AL

Behavioral outcomes were assessed using the OFT and SPT (Du Toit et al. 2020; Hatton‐Jones et al. 2022; Helman, Headrick, Peart, and Stapelberg 2022; Helman, Headrick, Vider, et al. 2022). Mice were first assessed in the OFT before undertaking a SPT 24 h later (limiting potential acute influences of the OFT on SPT outcomes). The OFT was conducted in a separate test room between 10:00 and 14:00 h. The test was undertaken in an 80 × 80 cm Perspex arena with 30 cm high walls. The floor was marked with a central 40 cm^2^ center square surrounded by a peripheral area. Mice were habituated to the room for a minimum of 30 min prior to placement in the OFT arena, at which point investigators left the room. The animals were recorded via digital video for 30 min within the OFT, after which the video recording was stopped and mice returned to their housing cages. The first 5 min of each video (Gould et al. 2009) was analyzed for behavioral measures including locomotor activity (line crossing/distance, velocity) and wall‐seeking behavior (number, duration) using EthoVision XT 14 (Nodulus Information Technology, Wageningen, Netherlands).

The SPT was performed in the same room that mice were housed in. Mice were placed into cages with ad libitum access to pure water and a 1% sucrose solution in separate bottles. Water and sucrose solution consumptions were recorded over 48 h, with the sucrose‐containing bottle switched from the right to the left of the cage in the second 24‐h period (to limit potential side preference bias). Mice were returned to housing cages at the conclusion of the SPT. Outcomes from both tests were incorporated into behavior subsystem AL scores.

### 2.6 Assessment of Metabolic and Neuro‐Endocrine‐Inflammatory ALs

For fasting blood measures, mice were fasted for 4 h before sampling via tail tip bleeds (between 0800 and 1130 h) 24 h after the SPT. Blood glucose was immediately measured with an Accu‐Check Performa glucose monitor, and additional blood was sampled for serum isolation. Non‐fasted blood was additionally collected at sacrifice. For serum analysis, samples were placed on ice for 30 to 60 min before 10 min centrifugation (2000 rpm), removal of serum, and storage at −80°C until analysis.

Fasted serum samples were assessed for glucose (as above), together with enzyme‐linked immunosorbent assay (ELISA) analysis of insulin, triglyceride, and cholesterol levels, according to manufacturer instructions (Ultra‐Sensitive Mouse Insulin ELISA Kit, Crystal Chem, Illinois, USA; Triglyceride Quantification Colorimetric/Fluorometric Kit, BioVision, California, USA; QuickDetect Total cholesterol (Mouse) ELISA Kit, Biovision, California, USA). Homeostatic model assessment of insulin resistance (HOMA‐IR) values were calculated for each mouse (Du Toit et al. 2020): [glucose] (mg/dL) × [insulin] (μU/mL)/405. Metabolic subs‐system AL scores were constructed from these measures, as outlined below.

For neuro‐endocrine‐inflammatory changes, non‐fasted blood was analyzed for leptin, noradrenaline, and the immuno‐inflammatory mediator IL‐6 via ELISA, according to manufacturer instructions (Mouse Leptin and Ghrelin ELISA kits, Cusabio, Houston, Texas; Norepinephrine ELISA Kit #KA3836, Abnova, Taipei, Taiwan). We additionally assessed melatonin levels in a subset of mice (Melatonin ELISA Kit #E4630, Milpitas, California), with results shown in Figure S2 of Data S1.

### 2.7 Assessment of Cardiovascular AL, Resilience and Adaptation

To detail cardiovascular AL and test for changes in intrinsic and adaptive resilience, hearts were isolated at the end of the study and Langendorff perfused for measurement of baseline contractile and coronary function, intrinsic resilience to I‐R insult, and the efficacy of adaptive protection via PC (Du Toit et al. 2020; Headrick et al. 2001; Peart and Headrick 2003). Mice were anesthetized with 60 mg·kg^−1^ sodium pentobarbital (I.P.) and hearts excised and immediately mounted on a Langendorff system. Hearts were perfused via the aorta at a pressure of 80 mmHg with modified Krebs–Henseleit solution (119 mM NaCl, 11 mM glucose, 22 mM NaHCO_3_, 4.7 mM KCl, 1.2 mM MgCl_2_, 1.2 mM KH_2_PO_4_, 1.2 mM EDTA, and 2.5 mM CaCl_2_) gassed with 95% O_2_ and 5% CO_2_ and maintained at 37°C. A fluid‐filled polyvinyl chloride film balloon was placed in the left ventricle via an incision in the left atrium and inflated to an end‐diastolic pressure (EDP) of 5 mmHg. Hearts were immersed in a jacketed bath of perfusion fluid at 37°C. A thermal probe connected to a Physitemp TH‐8 digital thermometer (Physitemp Instruments Inc., Clifton, NJ, USA) monitored perfusion fluid temperature throughout experiments. An ultrasonic flow probe proximal to the aortic cannula and connected to a T206 flowmeter (Transonic Systems Inc., Ithaca, NY, USA) continuously monitored coronary flow. Systolic pressure and EDP, heart rate, +d*P*/d*t* and ‐d*P*/d*t*, and coronary flow were recorded using a 4‐channel MacLab system (AD instruments Pty Ltd., Castle Hill, Australia) connected to an Apple iMac computer.

Intrinsic heart rate was measured after an initial 10 min stabilization period, before commencing ventricular pacing at 420 beats·min^−1^. After 25 min, baseline contractile function and coronary flow were measured. Hearts with substantially aberrant baseline function (excessive coronary flow, poor contractile development, according to criteria detailed previously (Reichelt et al. 2009)) were excluded from further study. Hearts were then subjected to 25 min normothermic global ischemia and 45 min aerobic reperfusion (to assess intrinsic resilience). Coronary effluent was collected on ice during postischemic reperfusion and stored at −80°C until determination of lactate dehydrogenase (LDH) levels via enzymatic assay, as detailed previously (Headrick et al. 2001; Peart and Headrick 2003; Peart et al. 2014). Total washout of cellular LDH indicates extent of myocardial damage during I‐R. We have previously demonstrated a good correlation between LDH efflux and myocardial infarction in this model (Peart and Headrick 2003). At the end of reperfusion, all hearts were removed into ice‐cold perfusion fluid, ventricular tissue dissected, and samples snap frozen in liquid N_2_ before storage at −80°C (for subsequent molecular analyses). Ventricular TNF‐α levels were measured as an indicator of postischemic inflammatory activity (see Ventricular Tissue Analyses below).

In order to test the influences of WD feeding and/or CS on myocardial adaptive responses, we examined the ability of prior exposure to brief non‐injurious I‐R (i.e., preconditioning) to increase ischemic tolerance. A subset of hearts (*n* = 8 per group) was conditioned with three episodes of 5 min ischemia/5 min reperfusion prior to the 25 min index ischemia. We have confirmed cardioprotective influences of this PC stimulus in this heart model (Russell et al. 2019; Russell et al. 2020).

### 2.8 Estimation of Subsystem and Systemic Allostatic Loads

Systemic AL was determined from loads across four critical physiological systems, each determined from multiple variables: *metabolic AL* (body weight, glucose, insulin, HOMA‐IR, triglycerides, and cholesterol); *neuro‐endocrine/immune AL* (noradrenaline, Il‐6, and leptin); *behavioral AL* (SPT and OFT outcomes); and *cardiovascular AL* (heart rate, coronary flow, contractility, and relaxation; and resilience determined from postischemic diastolic, contractile and coronary function, LDH efflux, and TNF‐α expression). Note that to permit calculation of individual ALs for all mice per experimental group, mean values for noradrenaline were substituted for two missing samples in each of the four groups.

The issue of variable weighting is fundamental to composite multivariate measures such as AL. A z‐score analysis, as in prior studies (Gillespie et al. 2019; Helman, Headrick, Peart, and Stapelberg 2022), makes no assumptions regarding relative weights of constituent variables and subsystems, and effectively normalizes parameters with diverse scales/units (according to variance from a control state). In contrast, early studies constructed the AL index by summating dichotomized scores for variables falling either below (scored 0) or within (scored 1) the highest risk quartile for the study population. In each approach, individual variables are equally weighted, though analyzed as continuous versus dichotomized measures, respectively. The former is preferred, and the latter is to be avoided where possible, as dichotomization of continuous variables reduces embedded information content, predictive power, and biological relevance (Royston et al. 2006; Salis et al. 2023; Taylor and Yu 2002). Additionally, subsystem (thus systemic) AL indexes from summated scores may be biased/skewed according to the number of variables measured. We focus on *z*‐score analysis—equally weighting individual variables within each subsystem, and contributions of each subsystem to systemic AL—and also calculate a dichotomized AL index for purposes of comparison.

### 2.9 Ventricular Tissue Analyses

Frozen left ventricular tissue was placed in a mortar filled with liquid N_2_ and homogenized with a pestle. Samples were homogenized in lysis buffer (97% cell lysis buffer, Bio‐Plex Cell Lysis Kit #171304011, Bio‐Rad, Australia; 2% Bimake Phosphatase Inhibitor Cocktail, Sapphire Biosciences, NSW, Australia; 1% Bimake Protease Inhibitor Cocktail (EDTA‐Free, 100X in DMSO), Sapphire Biosciences, NSW, Australia). Protein contents were determined via BCA assay, with absorbance measured at 540 nm on a Tecan Sunrise Microplate Reader with Magellan Standard software (TECAN, Grödig, Austria). Aliquots were then adjusted to fixed protein concentrations with Kinexus buffer (20 mM MOPS, 2 mM EGTA, 5 mM EDTA, 30 mM NaF, 40 mM β‐glycerophosphate, 20 mM NaPP, and pH 7.2), and stored at −80°C for later analysis.

Myocardial TNF‐α content was determined in homogenate samples via ELISA (TNF‐α Mouse AlphaLISA Detection Kit, PerkinElmer, Waltham, Massachusetts), and samples were subjected to proteomic interrogation via mass spectrometry. Sample volumes equivalent to 50 μg protein were transferred to chilled tubes, and MilliQ Ultrapure Water (Merck‐Millipore, Bedford, USA) added to normalize loading volumes. Reduction of each sample was completed using 100 mM dithiothreitol (Bio‐Rad Laboratories Inc., Hercules, USA) and 100 mM ammonium bicarbonate (Sigma Aldrich, Missouri, USA) in MilliQ water (1:1:3) at 37°C for 30 min, followed by alkylation using 200 mM iodoacetamide (Sigma Aldrich, Missouri, USA) for 15 min at room temperature. The pH of each sample was checked using pH indicator strips (Merck‐Millipore, Bedford, USA) and adjusted to pH 7–9 where necessary. Samples were buffer exchanged on Amicon 3 kDa spin filters (Merck‐Millipore, Bedford, USA) using 100 mM ammonium bicarbonate. The protein solution was digested with Sequencing Grade Modified Trypsin (Promega Corporation, Madison, USA) at 37°C for 16–18 h at a sample‐to‐trypsin ratio of 1:50. Each sample was transferred into a 1.5 mL tube with volume normalized using 10 mM ammonium bicarbonate. To stop the trypsin reaction, 1 μL of 99% formic acid was added to each sample, followed by a brief vortex and centrifugation at 21,300*g* for 5 min (ambient temperature). A 2 μL undiluted sample was transferred to an LC–MS vial and loaded onto the autosampler.

Digested peptides were separated by nanoLC using Ultimate3000 nano RSLC UPLC and an autosampler system (ThermoFisher, Illinois, USA). Samples were concentrated and desalted on a micro C18 pre‐column (300 μm x 5 mm, Dionex) with H_2_O:CH_3_CN (98:2, 0.1% TFA) at 15 μL/min. After a 4 min wash, the pre‐column was switched (Valco 10 port UPLC valve, Valco, Houston, TX) into line with a fritless nano column (75 μ × ~20 cm) containing C18AQ media (1.9 μm, 120 Å Dr. Maisch, Ammerbuch‐Entringen Germany). Peptides were eluted using a linear gradient of H_2_O:CH_3_CN (98:2, 0.1% formic acid) to H_2_O:CH_3_CN (64:36, 0.1% formic acid) at 200 nL/min over 50 min. High voltage (2000 V) was applied to a low volume Titanium union (Valco) with the column oven heated to 45°C (Sonation, Biberach, Germany) and the tip positioned ~0.5 cm from the heated capillary (T = 300°C) of a QExactive Plus (Thermo Electron, Bremen, Germany) mass spectrometer. Positive ions were generated by electrospray, and the QExactive operated in data‐dependent acquisition mode. A survey scan *m*/*z* 350–1750 was acquired (resolution = 70,000 at *m*/*z* 200, with an accumulation target value of 1,000,000 ions) and lock‐mass enabled (*m*/*z* 445.12003). Up to the 10 most abundant ions (> 80,000 counts, underfill ratio 10%) with charge states > +2 and < +7 were sequentially isolated (width *m*/*z* 2.5) and fragmented by HCD (NCE = 30) with an AGC target of 100,000 ions (resolution = 17,500 at *m*/*z* 200). The *m*/*z* ratios selected for MS/MS were dynamically excluded for 30 s.

### 2.10 Bioinformatic Analysis

Peak lists were generated using Mascot Daemon combined with Mascot Distiller, and submitted to an in‐house Mascot database server (Matrix Science; version 2.5.1). Data processing of raw data files was performed on MaxQuant (version 2.1.3.0) (Tyanova et al. 2016). Search parameters were as follows: +/−4.0 ppm tolerance for precursor ions; carbamidomethyl as a fixed parameter, and acetylation and oxidation as variable parameters. Data were searched against the Uniprot mouse database (downloaded on August 29, 2022). Differential protein abundance was performed in Perseus (Version 2.0.6.0). For each comparison, protein fold changes were determined for proteins with assigned label‐free quantification (LFQ) values. The *p*‐values associated with each log_2_ protein fold change were calculated using one sample t‐tests. Pathway enrichment analysis was completed on differentially expressed proteins using the QIAGEN Ingenuity Pathway Analysis (IPA) software. Proteins were regarded as differentially expressed based on both *t*‐test *p*‐values (*p* ≤ 0.05) and a fold change threshold (≥ 1.2 for upregulation and ≤ 0.83 for downregulation). Briefly, the gene identifiers and their fold change for FC and HPC samples of each experimental group were separately uploaded into the software. Analyses involved assessing the influences of: (i) diet (CD vs. WD); (b) stress (CD vs. CD + CS); and (c) stress in WD mice (WD vs. WD + CS). Significant interaction networks (*p* ≤ 0.05) and molecular and cellular functions were identified on known protein–protein interactions from published literature. All relevant data are available in the paper and Supporting Information.

### 2.11 Statistical Analysis

Statistical analysis was undertaken using XLSTAT (version 2024.3.0) in Microsoft Excel for Mac (V 16.91). For normally distributed parametric data, multiway ANOVAs were performed, with a REGWQ post hoc test where significant effects were detected (*p* < 0.05). Where data were not normally distributed (according to the Shapiro–Wilks test), Box‐Cox or Johnson data transformation was applied, and where this did not successfully normalize distributions (in this case, for circulating cholesterol and cardiac ‐dP/dt) the data were analyzed by nonparametric Kruskal–Wallis test with Dunn's post hoc test (Bonferroni corrected). Since AL indexes calculated from dichotomized data represent ordinal scores, these data were analyzed via Kruskal–Wallis test. Analysis of adaptive resilience (the PC responses in Figure 4) involved a planned comparisons design, testing a priori that: the PC stimulus adaptively increases I‐R resilience in either CD, CS, WD, or WD + CS hearts (–PC vs. +PC within each group); and resilience is altered by a WD, CS, or WD + CS (–PC between each group; +PC between each group). In all statistical tests, the α level was set to 0.05.

## 3 Results

### 3.1 Phenotypic Influences of a WD and CS: Metabolic, Neuro‐Endocrine‐Immune, and Behavioral SubSystem Outcomes

Subsystem outcomes support the emergence of a pre type 2‐diabetic phenotype with the WD (increased weight, insulin levels and resistance) coupled with evidence of anhedonia. The CS protocol appears to exert some beneficial metabolic effects and induces anxiety‐like behavior. Comorbid WD and CS thus produce cardinal features of depressive disorder, including anhedonia, anxiogenesis, and a relative weight loss (vs. CD or WD alone).

#### 3.1.1 Metabolic

**FIGURE 1:**
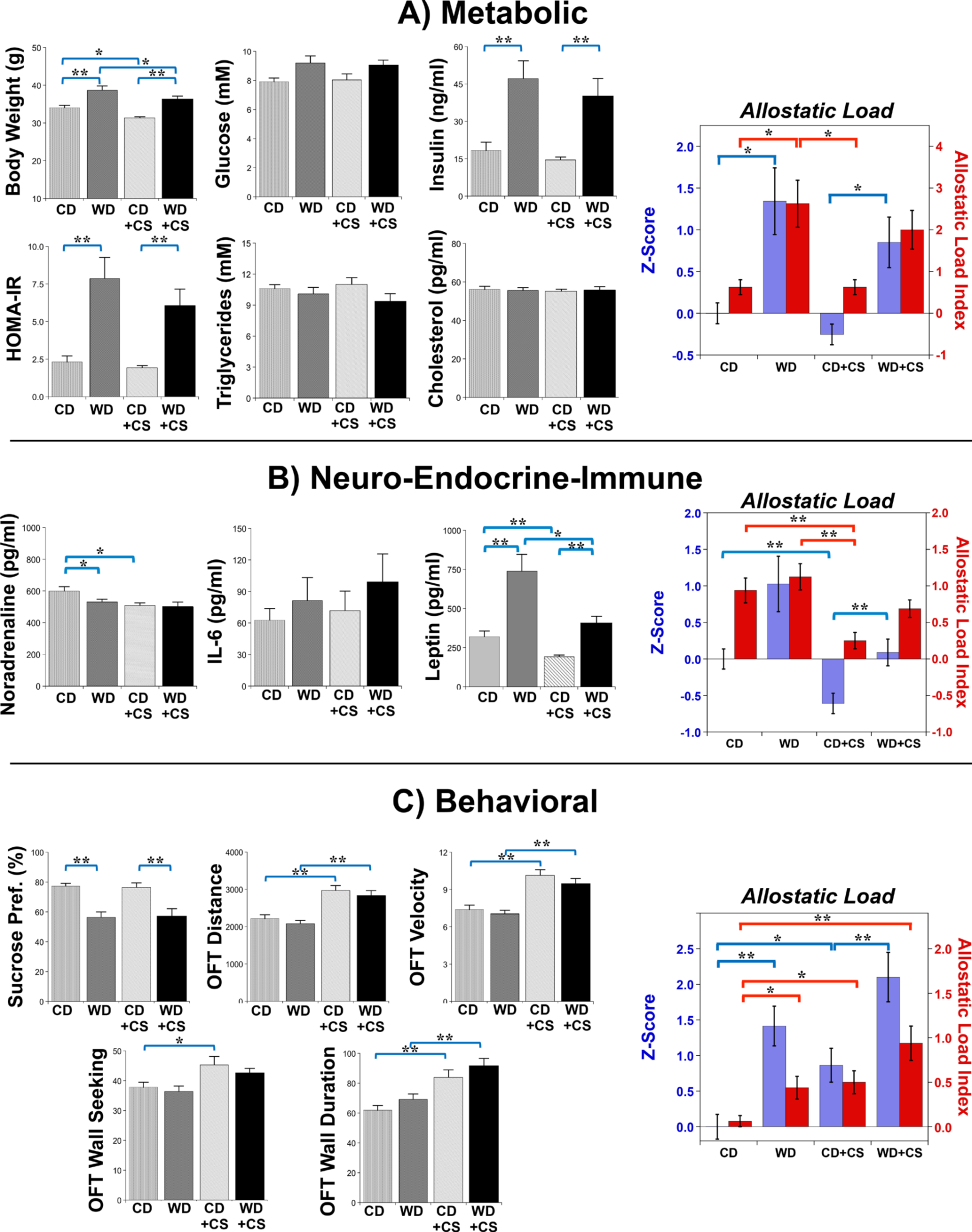
Effects of stress and diet on elements of the metabolic, neuro‐endocrine‐immune and behavioral subsystems, and associated allostatic load (AL) estimated via *z*‐score or dichotomization analysis. Data are means ± SEM (*n* = 16/group). Bars highlight significant differences between groups (*p* < 0.05; two‐way ANOVA, REGWQ test; with the exception of cholesterol, analyzed via Kruskal–Wallis and Dunns post hoc test; *p < 0.05, **p < 0.01).

The WD resulted in significant weight gain (Figure 1A; Figure S1), linked to higher daily caloric intake (Figure S1). Imposition of CS reduced body weight by ~4 g over 2 weeks in both CD and WD mice, an effect not linked to a consistent decline in calorie intake (10% reductions in weight with CS in CD and WD mice not associated with significant changes in caloric intake; Figure S1). Fasting blood glucose was not significantly elevated with the WD or CS (Figure 1A). Insulin levels and resistance were substantially increased by the WD and unaffected by CS. Mean triglyceride and cholesterol levels were not modified with either the WD or CS (Figure 1A). Overall, the metabolic phenotype with WD feeding resembles a pre type‐2 diabetic state: evolving obesity, hyperinsulinemia, and insulin resistance prior to the development of chronic hyperglycemia and dyslipidemia. As a result of these outcomes, metabolic AL was significantly increased with WD feeding but not CS, with no evidence of additive influences of the WD and CS (Figure 1A). Allostatic load changes were similar for both z‐score and dichotomous scoring approaches to estimation of AL.

#### 3.1.2 Neuro‐Endocrine‐Immune

Mean circulating noradrenaline levels were reduced with WD feeding and with CS, an effect not exaggerated by comorbid CS and WD feeding (Figure 1B). Circulating IL‐6 was not significantly modified, while leptin levels increased more than twofold with the WD and were reduced by CS (Figure 1B). This WD‐dependent elevation in leptin was significantly attenuated by comorbid CS. We additionally assessed melatonin in subsets of mice, which was unchanged with either the WD or CS (Figure S2). As a result of these variable responses, neuro‐endocrine‐immune AL remained relatively unchanged with the WD, whereas CS somewhat unexpectedly reduced neuro‐endocrine‐immune AL. As a result of these opposing actions, comorbid WD feeding and CS did not significantly modify neuro‐endocrine‐immune AL (Figure 1B).

#### 3.1.3 Behavioral

Sucrose preference was reduced by the WD (Figure 1C), a response unaltered by CS (which had no independent influence). The WD did not modify OFT measures of locomotion or wall‐seeking (Figure 1C). However, CS increased locomotion (distance and velocity) and wall‐seeking duration in CD and WD mice, together with wall‐seeking number in CD mice. Allostatic load for the behavioral subsystem was increased by both the WD and CS, with some evidence for additive influences: the behavioral AL from *z*‐score analysis of WD + CS mice was 241% and 150% of values for CS or WD alone; and the AL index from dichotomous scoring was 190% and 216% of the values for CS or WD alone (Figure 1C). However, there was no statistical difference between the AL score for WD + CS versus WD mice, while it was significantly higher than in CD and CD + CS mice.

### 3.2 Cardiovascular Responses—Function, Resilience, and Adaptation

**FIGURE 2:**
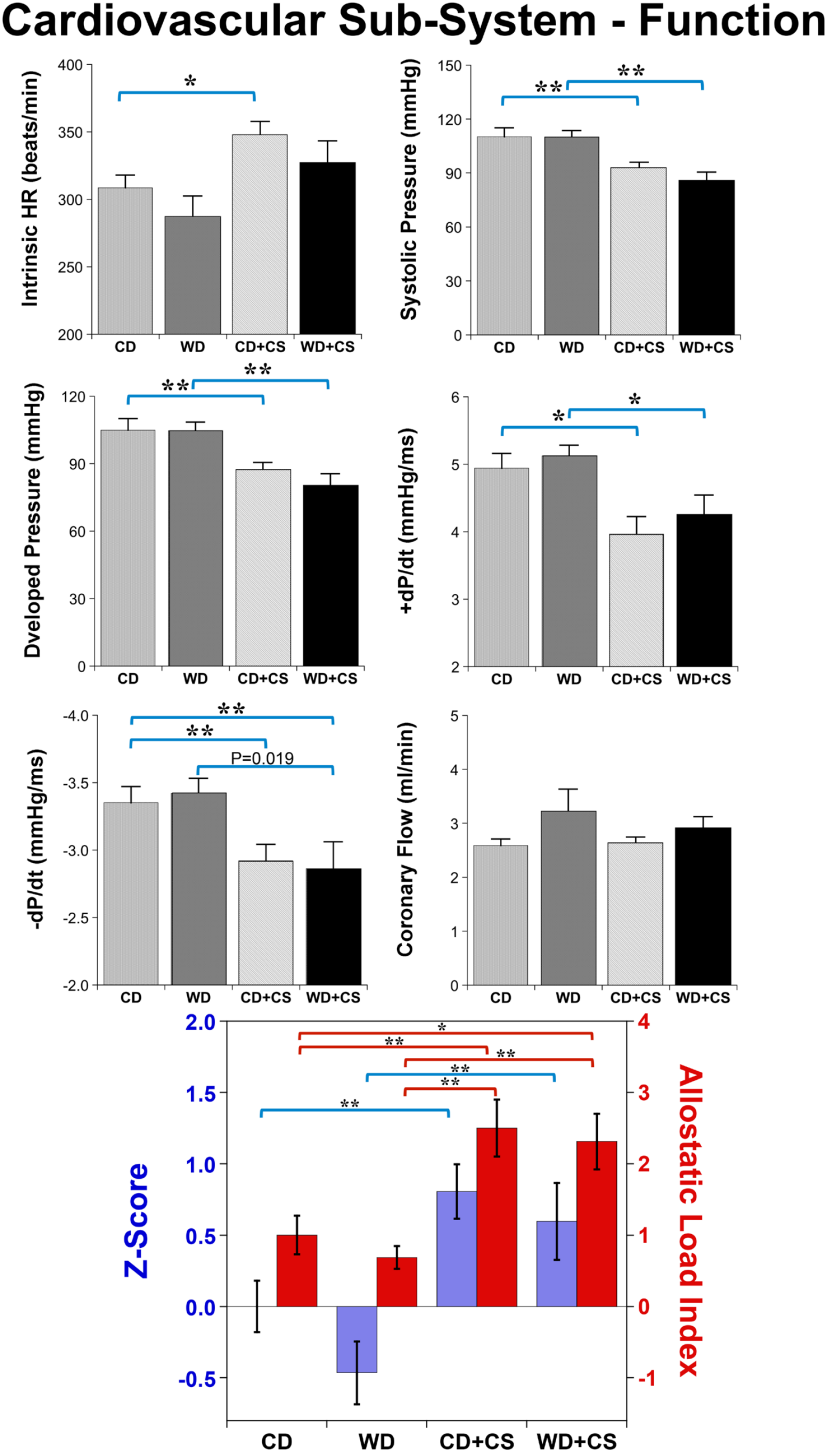
Effects of stress and diet on cardiac and coronary functional parameters, and cardiovascular functional allostatic load (AL). Data are means ± SEM (*n* = 16/group). Bars highlight significant differences between groups (*p* < 0.05; two‐way ANOVA, REGWQ post hoc test; with the exception of ‐dP/dt, analyzed via Kruskal–Wallis and Dunns post hoc test; *p < 0.05, **p < 0.01).

**FIGURE 3:**
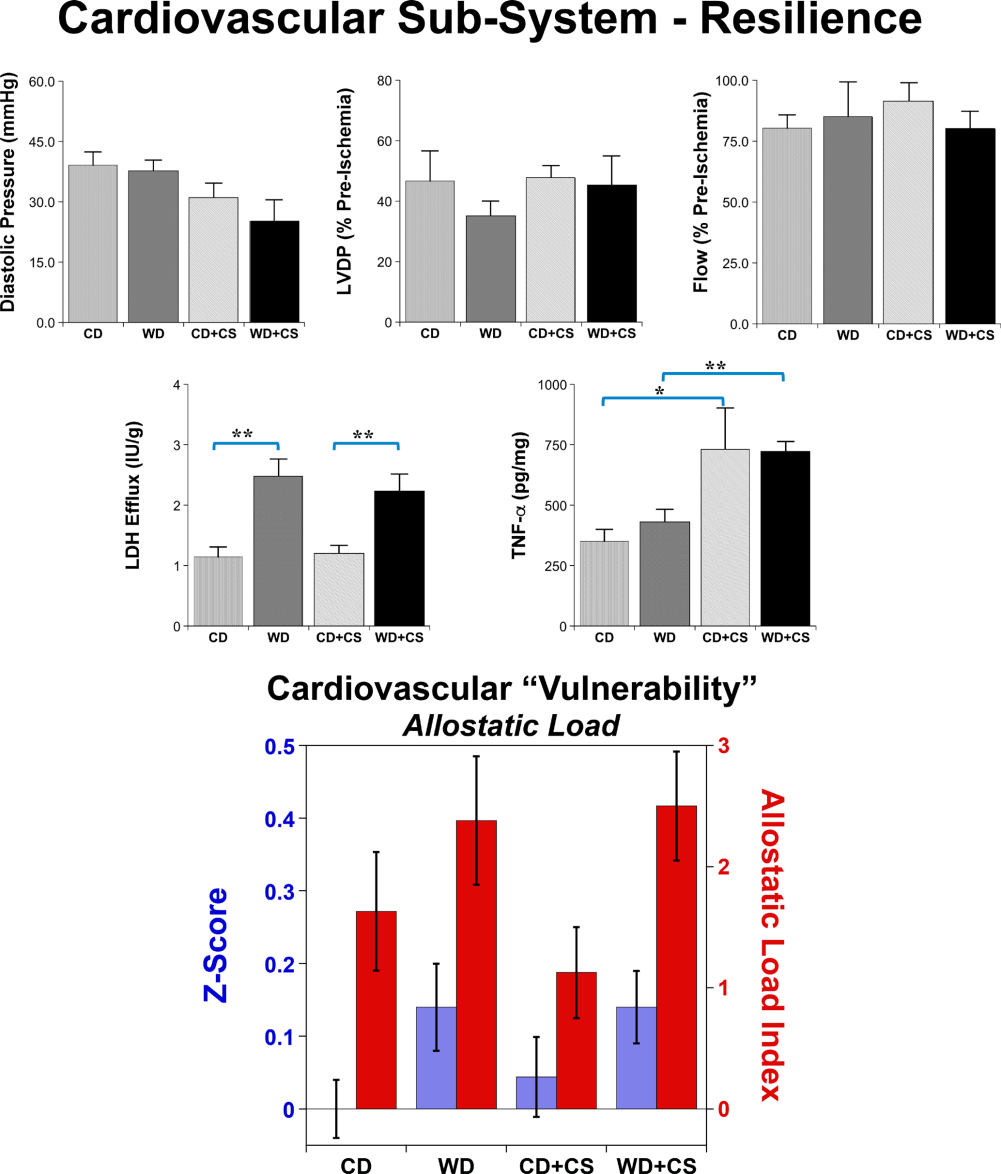
Effects of stress and diet on elements of cardiovascular resilience (or conversely vulnerability) and resilience allostatic load (AL). Measures include functional outcomes, cell death (LDH efflux) and pro‐inflammatory activity (TNF‐α). Data are means ± SEM (*n* = 8/group). Bars highlight significant differences between groups (*p* < 0.05; two‐way ANOVA, REGWQ post hoc test; *p < 0.05, **p < 0.01).

Contrasting effects across other subsystems, the WD had limited influence on the cardiovascular system, selectively reducing intrinsic resilience to I‐R (Figures 2 and 3). On the other hand, CS had multiple detrimental impacts, increasing intrinsic beating rate/automaticity in CD hearts (Figure 2A)—with a similar yet insignificant trend (*p* = 0.146) in WD hearts—and decreasing contractility (systolic and developed pressures, +dP/dt) in hearts from both diet groups (Figure 2B–D). Lusitropic state (indicated by –dP/dt) was also significantly reduced by CS (Figure 2E). Coronary flow (or resistance) was not modified with WD feeding or CS (Figure 2F). These intrinsic functional profiles in intact hearts (removed from nervous and endocrine influences) support significant functional disruption and increased “functional” AL with CS but not WD feeding, an effect not exaggerated in hearts from WD + CS mice (Figure 2).

In terms of intrinsic resilience, functional outcomes from I‐R were not significantly modified with either the WD or CS (Figure 3). However, myocardial injury indicated by coronary LDH efflux was increased with the WD, an effect unaltered by comorbid CS (Figure 3D). On the other hand, postischemic myocardial levels of the pro‐inflammatory cytokine TNF‐α were specifically increased by CS, an effect comparable in hearts from both diet groups (Figure 3E). These select influences did not result in a significant change in resilience related AL (index of overall vulnerability) with either the WD or CS (Figure 3).

**FIGURE 4:**
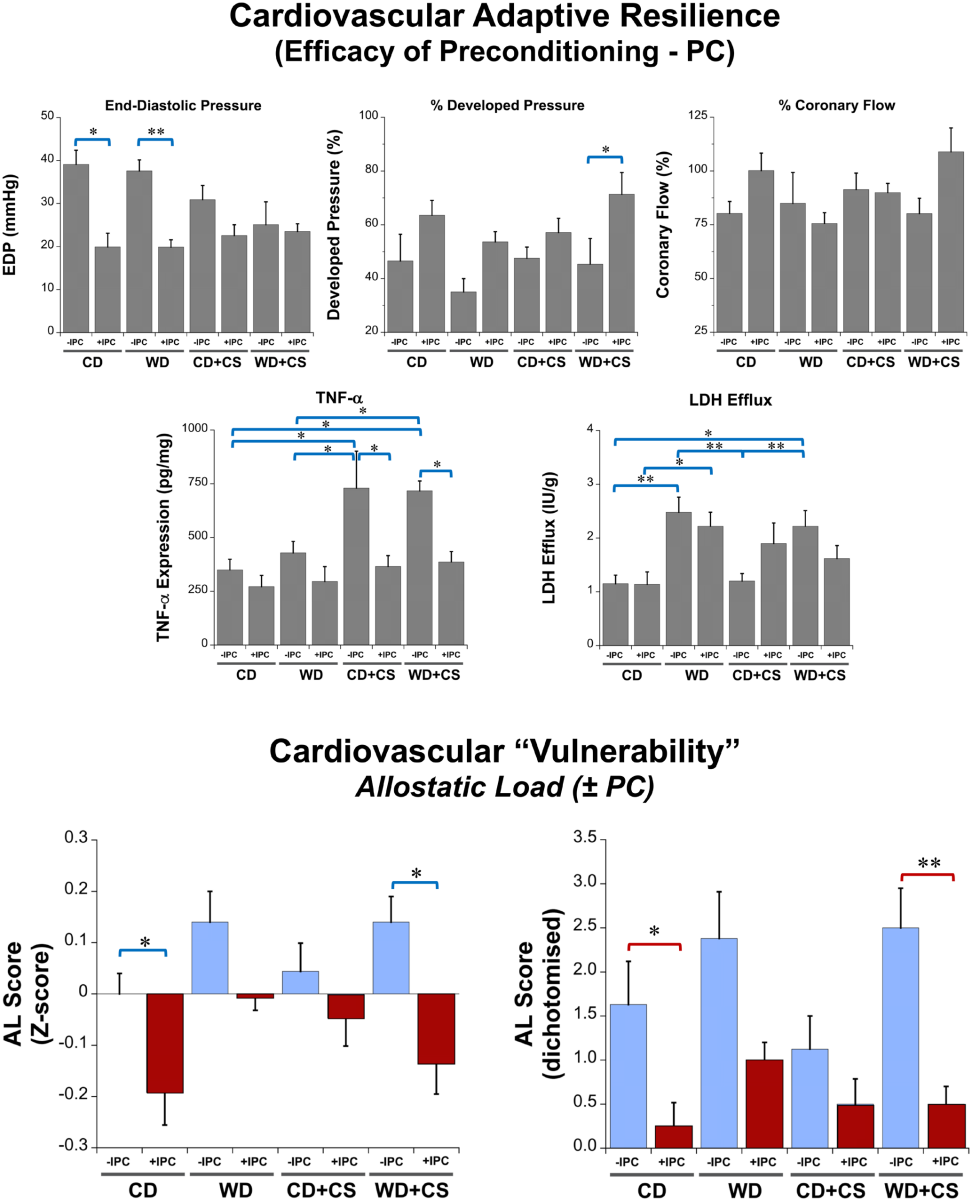
Analysis of adaptive resilience (PC) in hearts from CD and WD mice ± CS, together with estimates of resilience allostatic load (or cardiovascular vulnerability). Data are means ± SEM (*n* = 8/group). Bars highlight significant differences between groups (*p* < 0.05; two‐way ANOVA, REGWQ post hoc test; *p < 0.05, **p < 0.01).

Analysis of adaptive resilience—assessed from the ability of exposure to non‐injurious I‐R to enhance subsequent ischemic tolerance (i.e., preconditioning) ‐ confirms significant benefits in hearts from CD, WD, and also WD + CS mice (Figure 4). The PC stimulus reduced diastolic dysfunction in CD and WD hearts, improved pressure development in WD + CS hearts, and effectively countered the CS dependent elevation in postischemic TNF‐α, normalizing TNF‐α levels across groups (Figure 4). Cell death (from LDH efflux) did not appear influenced by PC in the present model (indeed, LDH efflux was insignificantly increased by PC in CD + CS hearts). These data indicate protection against postischemic dysfunction is negated by CS in CD hearts, yet appears preserved in WD + CS hearts (Figure 4).

We estimated overall resilience‐related AL (indicative of cardiac vulnerability) in both non‐PC and PC hearts, incorporating measures of diastolic dysfunction/stiffness, contractility, coronary function, cell death (LDH efflux), and inflammation (TNF‐α) in the score (Figures 3 and 4). This measure illustrates the distinct influences of the WD and CS on intrinsic and adaptive resilience. Neither the WD nor CS independently modified resilience‐related AL, and the PC stimulus reduces resilience AL in both CD and WD + CS hearts (protection being largely negated in hearts from CS mice) (Figures 3 and 4).

### 3.3 Influences of Diet and Stress on the Cardiac Proteome

**FIGURE 5:**
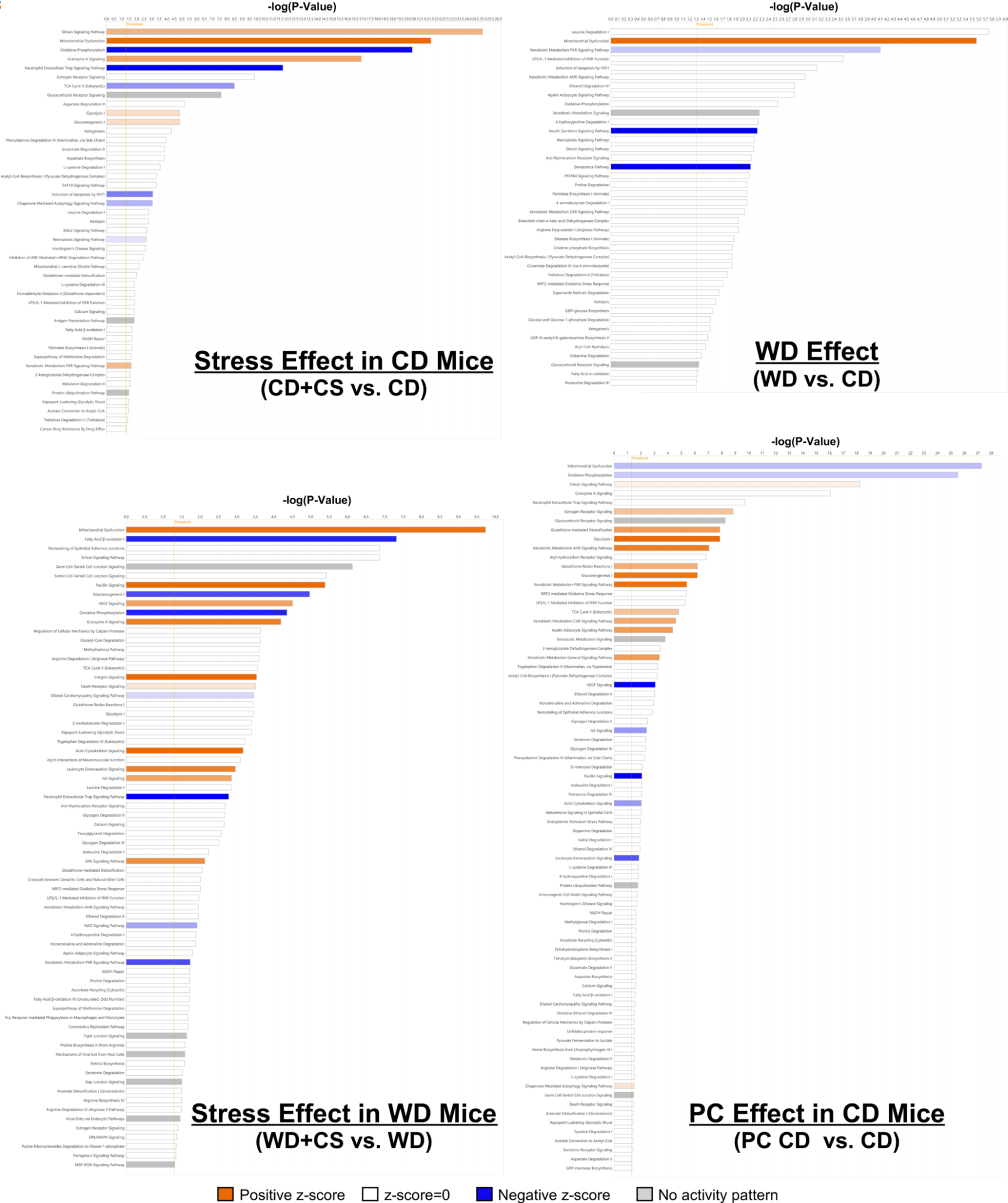
Canonical pathways modified by a WD, CS, or their combination; and by adaptive PC in CD mice.

Approximately 1900 proteins were identified in LV tissue, across CD (1643), WD (1543), CD + CS (1438), and WD + CS (1397) groups (Figures S3–S5, Table S1). Analysis of differentially expressed proteins (DEPs) indicates: main effect of CS in CD mice = 121 proteins; main effect of WD in unstressed mice = 48 proteins; and main effect of CS in WD mice = 117 proteins (Figures S3–S5, Table S1). A summary of the most significant pathway changes (from bioinformatic analysis—see below) with the WD, CS, combined WD + CS, and PC in CD mice is provided in Figure 5. Mitochondrial and immune/cell defense pathways were overrepresented in all groups.

#### 3.3.1 Effects of CS

Stress in CD mice modified proteins linked to mitochondrial elements and respiration/ATP generation, together with TCA cycle, gluconeogenesis, fatty acid, and other energy metabolism related pathways (Figure S3). The strongest pathway responses to CS included: upregulation of proteins involved in sirtuin signaling, mitochondrial dysfunction, and granzyme A signaling; and downregulation of proteins involved in oxidative phosphorylation and neutrophil extracellular trap signaling (Figure S3). Other changes included increased glycolysis and gluconeogenesis, and decreased TCA cycle proteins.

#### 3.3.2 Effects of WD Feeding

The WD also strongly modified proteins linked to mitochondria, with a greater influence on lipid/fatty acid metabolism proteins (Figure S4). The strongest pathway responses to WD feeding involved increased mitochondrial dysfunction proteins and reductions in xenobiotic metabolism PXR signaling, insulin secretion signaling, and senescence pathway related proteins (Figure S4). Other changes included increased glycolysis and gluconeogenesis proteins and a reduction in TCA cycle proteins.

#### 3.3.3 Effects of Combined CS in WD Mice

Effects of stress differed in hearts from WD versus CD mice. While CS again primarily influenced proteins associated with mitochondria, CS in WD mice additionally influenced sarcomeric, Z‐disk, fascia adherens, and cytoskeletal proteins (Figure S5), suggesting a unique influence of stress on sarcomeric and intercalated disk structure/function in the hearts of WD mice. Functionally, these proteins were most strongly linked to actin binding, cytoskeletal structure, and other protein binding processes (Figure S5). The most significant pathway responses to stress in WD mice involved increases in mitochondrial dysfunction, paxillin, granzyme A, VEGF, integrin, death receptor, actin cytoskeleton, leukocyte extravasation, and ILK signaling proteins; and reductions in fatty acid ß‐oxidation, gluconeogenesis, oxidative phosphorylation, and neutrophil extracellular trap signaling proteins (Figure S5).

#### 3.3.4 Response to Cardiac PC

The adaptive PC response involves some protein changes that are distinct or opposed to those arising with CS or WD feeding (Figures S6–S10). In CD hearts, PC most significantly modified mitochondrial/respiratory chain together with *z*‐disk and sarcomere proteins (Figure S6). Functionally, ATP synthesis/respiration and muscle contraction/sarcomere organization were the most modified. Pathway responses exhibited a trend to downregulation of both mitochondrial dysfunction and oxidative phosphorylation proteins, together with: reductions in VEGF, ILK, paxillin, and actin cytoskeleton signaling proteins; and increases in regulation of estrogen receptor, glutathione‐mediated detoxification, glycolysis, xenobiotic metabolism (PXR, CAR, and general signaling), TCA cycle, and apelin adipocyte signaling proteins (Figure S6).

The PC stimulus did not induce functional protection in hearts from CD + CS mice, though it did reduce TNF‐α levels. Pathway responses in CD + CS hearts indicate that PC increased oxidative phosphorylation, neutrophil extracellular trap signaling, TCA cycle, estrogen receptor signaling, and xenobiotic metabolism AHR/PXR/CAR signaling proteins; and reduced mitochondrial dysfunction, sirtuin signaling, granzyme A, aspartate degradation, leukocyte extravasation, and macrophage classical activation pathway proteins (Figure S7).

Direct comparison of PC responses in CD + CS versus CD hearts indicates: a relative upregulation of oxidative phosphorylation, RhoGDI, and sirtuin signaling proteins; and a relative downregulation of mitochondrial dysfunction, actin cytoskeleton, ILK, RHOA, Rho family GTPase, leukocyte extravasation, and integrin signaling proteins. This suggests a decline in cytoskeletal/cell‐ECM interactions and immune cell function proteins in PC hearts from stressed versus unstressed mice, together with increased expression of oxidative phosphorylation and sirtuin signaling proteins, and disturbed Rho family GTPase signaling.

In hearts from WD mice, the PC stimulus: increased oxidative phosphorylation, sirtuin, granzyme A, estrogen receptor, neutrophil extracellular trap, immunogenic cell death, and xenobiotic metabolism AHR and PXR signaling proteins, together with TCA cycle, glycolysis, and gluconeogenesis proteins; and reduced mitochondrial dysfunction, together with leukocyte extravasation signaling and macrophage classical activation signaling proteins (Figure S8). Consistent with a preserved efficacy of PC in WD hearts, there were minimal pathway differences in PC hearts from WD versus CD mice, with greater expression of mitochondrial dysfunction and sirtuin signaling proteins the only evident differences (Figure S8). In WD + CS hearts, the PC stimulus increased oxidative phosphorylation proteins, dilated cardiomyopathy, neutrophil extracellular trap, RhoGDI, and xenobiotic metabolism AHR signaling proteins, and proteins associated with the TCA cycle, glycolysis, and fatty acid ß‐oxidation; and reduced mitochondrial dysfunction, ILK, actin cytoskeleton, leukocyte extravasation, integrin, VEGF, paxillin, and RhoA/Rho GTPase family/Rho regulation of actin motility, cardiac hypertrophy, and macrophage classical activation signaling proteins (Figure S9).

Summaries of responses to CS, the WD, comorbid WD + CS, and PC in each of these groups are provided in Figure 5 and Figure S10. Overall, protein profiles indicate that PC modifies mitochondrial function, cell–ECM interaction, and inflammatory cell signaling/associated apoptosis, xenobiotic metabolism pathways, glutathione antioxidant defenses, carbohydrate metabolism (glycolysis, TCA) and cardioprotective estrogen signaling (Figure 5 and Figure S10).

#### 3.3.5 Mitochondrial Dysfunction Pathway Responses

**FIGURE 6:**
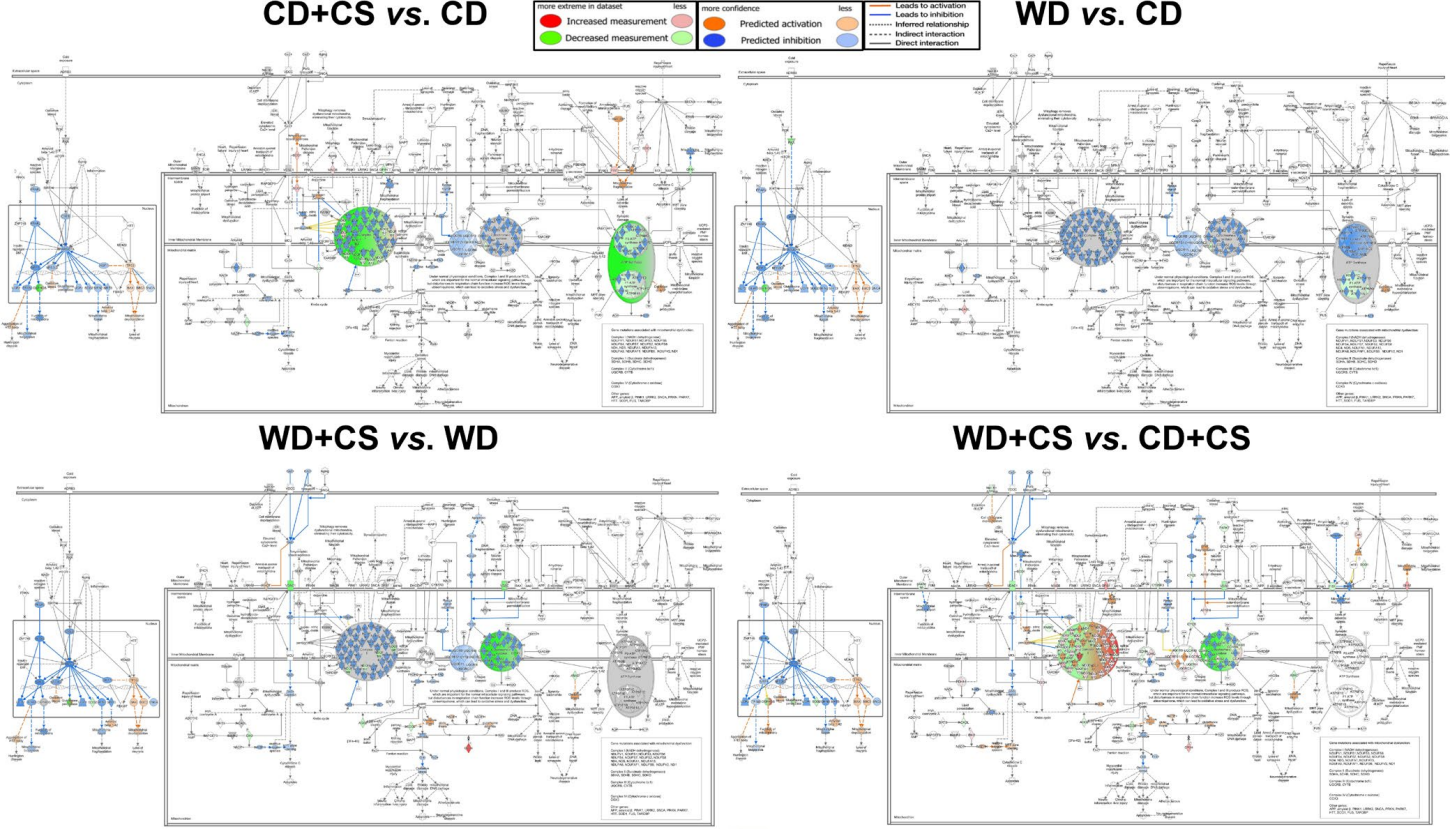
Influences of stress and diet on the myocardial mitochondrial dysfunction path. Shown are effects of stress (CD + CS vs. CD), the WD (WD vs. CD), and stress in WD mice (WD + CS vs. WD), together with differences in stressed CD versus stressed WD mice (WD + CS vs. CD + CS).

Focusing on highly represented mitochondrial processes, details of WD and/or CS effects on the mitochondrial dysfunction pathway are provided in Figure 6. Stress alone (in CD mice) suppressed CI, CIII, CIV, and ATP‐synthase activities (predicted to reduce ATP generation). Stress also decreased the activity of key nuclear signaling (including PPARγ, CREB, and PPARGC1A; and downstream targets/mediators NRF1, UCP1, UCP2, glutathione peroxidase, TFAM SOD2, MFN2, and SIRT3). This is predicted to suppress mitochondrial biogenesis, oxidative phosphorylation, and fusion. Stimulatory effects on TP53, BAX, BBC3, and amyloid beta 1‐42 are predicted to increase oxidative stress, mitochondrial depolarization, and cell death. In addition, effects on TARDEP, FIS1, SOD1, DNM1L, and OPA1 are predicted to favor mitochondrial dysfunction and fragmentation versus fusion. The WD alone had almost identical influences on nuclear signaling and also suppressed CI, CIII, CIV, and ATP synthase activities (without shifts in fission/fusion mediators). Stress in WD mice reduced CI, CIII, and CIV activities and subunit expression, with little influence on ATP‐synthase. Interestingly, VDAC1 was selectively reduced and GPX4 increased by CS in WD mice. Nuclear signaling was broadly suppressed in WD + CS versus CD + CS hearts, with pro‐death signaling via TP53/BAX BBC3, and oxidative stress apparently upregulated (Figure 6).

Comparing WD + CS to CD + CS reveals a mix of effects on CI (both up‐ and downregulation), reduced CIV activity, and no further influence on ATP‐synthase (Figure 6). Nuclear signaling is broadly suppressed in WDCS versus CDCS hearts, with TP53/BAX BBC3 and ox stress upregulated. Overall, these data support inhibitory influences of stress on CI, CIII, CIV, and ATP synthase activities and nuclear signaling (impairing biogenesis, and enhancing depolarization, apoptosis, and oxidative stress), while favoring a shift toward mitochondrial fragmentation. The WD alone induces similar, albeit less pronounced, effects on respiratory complexes and nuclear signaling, while comorbid stress in WD mice has further inhibitory influences on CI and CIV activities and nuclear signaling.

### 3.4 Cardiovascular and Systemic AL

**FIGURE 7:**
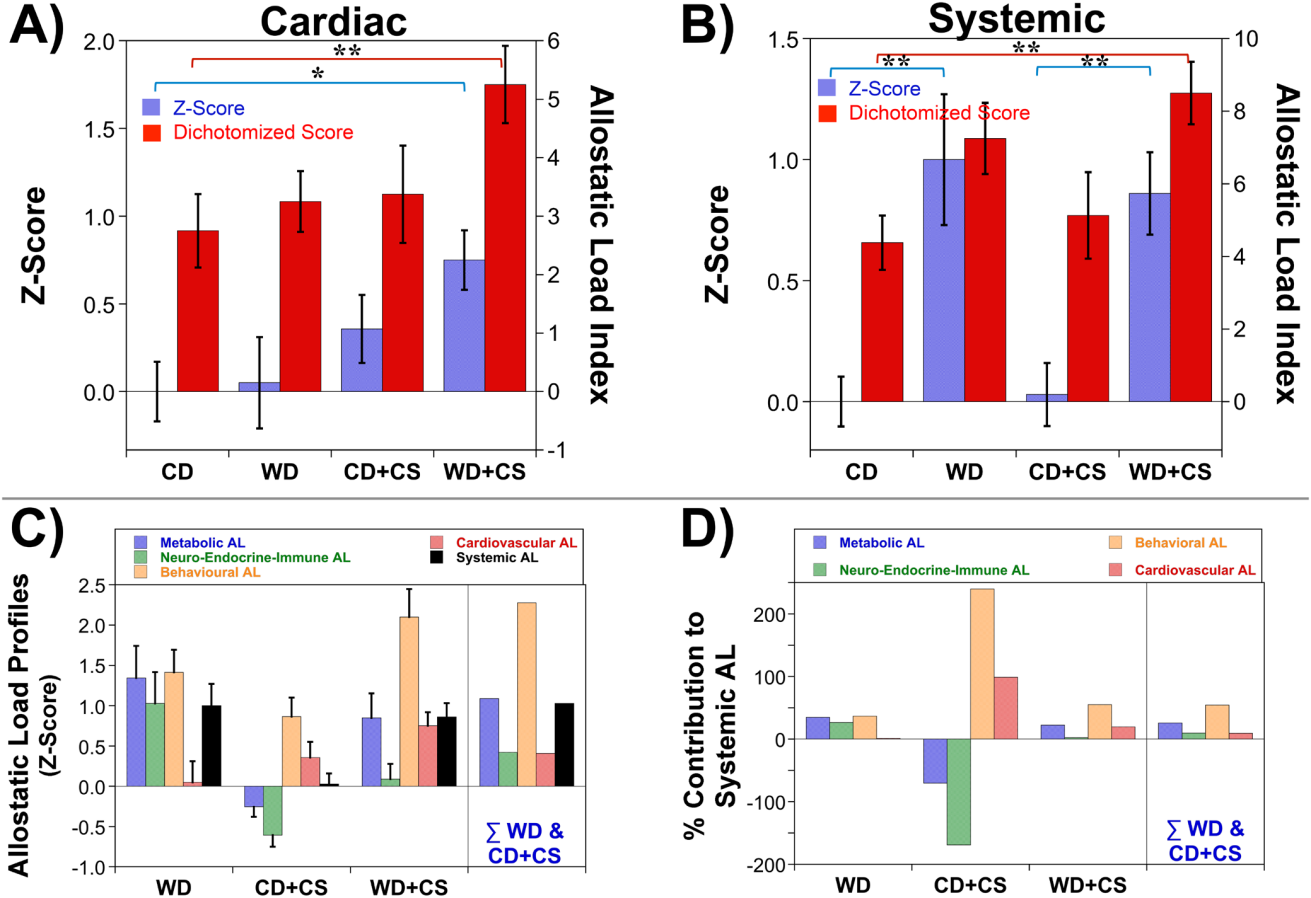
Overall cardiovascular and systemic allostatic loads (ALs). The cardiovascular AL (A) incorporates both functional measures and measures of intrinsic resilience. Scores for each subsystem (metabolic, neuro‐endocrine‐immune, behavioral, and cardiovascular) are combined to provide a meta or systemic AL (B). Data are means ± SEM (*n* = 16/group). Bars highlight significant differences between groups (*p* < 0.05; two‐way ANOVA, REGWQ post hoc test for *z*‐score AL; Kruskal–Wallis test for dichotomous AL index’ *p < 0.05, **p < 0.01). Lower panels depict the AL profiles in terms of absolute values (C) or % contributions (D) to systemic AL. Also shown to the right of data for WD + CS mice are AL values estimated by mathematically summating effects of individual WD and CS (i.e., profiles predicted based on additive influences of the WD and CS).

The overall cardiovascular AL (combining functional and resilience measures) and systemic AL are shown in Figure 7. Cardiovascular AL was not modified by either a WD or CS individually; however, combined WD + CS significantly increased this index (Figure 7A). Data thus support additive cardiovascular influences of comorbid WD feeding and CS. Combining this measure of AL with those for metabolic, behavioral, and neuro‐endocrine‐immune subsystems provides an estimate of systemic AL (Figure 7B). This index of systemic AL was increased with WD feeding but not CS, with no evidence of any additive influences of the two stressors (Figure 7B).

The AL profiles for WD, CS, and WD + CS mice, including absolute and % contributions of subsystems to the systemic load, are summarized in Figure 7C,D. These figures highlight distinct influences of WD and CS on subsystem and systemic loads: WD‐dependent AL involves equal contributions from metabolic, neuro‐endocrine‐immune and behavioral subsystems; CS‐dependent AL involves increased behavioral and cardiovascular load (vs. reductions in metabolic and neuro‐endocrine‐immune ALs); and behavioral AL is the dominant contributor to systemic load in comorbid WD + CS mice (with lesser contributions from metabolic and cardiovascular loads). Predicted profiles derived by mathematically summating individual subsystem loads for WD and CS mice are shown for comparison (right‐most panels in Figure 7C,D). Agreement between values determined in this way and those measured in WD + CS mice is consistent with additive interactions between diet and stress in determining metabolic, behavioral, and systemic AL (Figure 7C,D). On the other hand, the higher cardiovascular AL in WD + CS mice compared with the predicted value suggests a greater than additive (potentially synergistic) influence of comorbid WD + CS on cardiovascular AL.

In terms of validating measures of AL, linear regression confirms limited correlation between different elements of AL (Figure S11). This is predicted from fundamental principles: (i) that allostasis involves complex, sometimes opposing changes within and across physiological subsystems; (ii) that no individual (or correlated) variable can capture this complex integrated response; and (iii) that AL is therefore only assessable from analysis of multiple biological variables and systems. That said, select biological variables did exhibit correlations, notably elements within the metabolic subsystem: positive correlations were evident between body weight, insulin, HOMA‐IR, and leptin (Figure S11). The two different measures of AL (*z*‐score, dichotomized data) were also positively correlated, supporting interchangeability (though the two approaches yield select differences in limited instances).

## 4 Discussion

The present study confirms key predictions of allostasis, adaptive homeostasis, and reactive scope models, including stress‐dependent impairment of system functionality, resilience, and adaptation, in association with impairments in energy metabolism and defense processes. Chronic dietary and psychosocial stressors disrupt cardiovascular, behavioral, and metabolic systems in both an independent (unique cardiovascular/behavioral abnormalities, and opposing metabolic outcomes) and interactive or additive manner (comorbidity necessary to increase cardiovascular AL and induce a depression‐like state). These observations collectively support co‐development of cardiovascular and affective disorders (in particular) as a result of additive interactions between two common environmental stressors. We discuss these findings in the context of the three questions addressed: (i) do psychosocial and metabolic stressors interact to impair function, resilience, and adaptation; (ii) what molecular pathways are involved; and (iii) how do psychosocial and dietary stress interact to influence individual and systemic allostatic loads.

### 4.1 Stress and a WD Impair Cardiovascular Function, Resilience and Adaptation

Allostasis, adaptive homeostasis, and the related reactive scope model predict a progressive decline in functionality, resilience, and adaptability as a result of stress‐dependent “wear and tear,” ultimately promoting aging and chronic disease development. We have confirmed such changes with aging (Ashton et al. 2023; Peart et al. 2014), and results here support similar dysfunction with comorbid CS and a WD. The heart appears particularly vulnerable to CS, which disrupted automaticity, inotropy, lusitropy, postischemic inflammatory activity, and adaptive protection. In contrast, the WD selectively reduced intrinsic resilience. Importantly, only a combination of CS in WD mice resulted in a significant increase in cardiovascular AL, supporting pathological additivity between these risk factors.

#### 4.1.1 Stress‐Dependent Cardiac Dysfunction

Chronic stress has been shown to increase resting heart rate in animal models (Duarte et al. 2015; Grippo et al. 2002; Mercanoglu et al. 2008), attributed in part to altered autonomic control (Grippo et al. 2002). The present data support increased cardiac automaticity, evident in isolated myocardium. Restraint stress reportedly increases cardiomyocyte L‐type Ca^2+^ channel expression and activity (Zhao et al. 2009), and modifies myocardial connexin‐43 expression and gap junction function (Grippo et al. 2015), influencing intrinsic electrophysiology. Maladaptive hypertrophy and fibrosis may additionally modify refractoriness and automaticity (Carnevali et al. 2013; Costoli et al. 2004). A stress‐dependent increase in resting heart rate may not only increase energy costs of cardiac work and limit cardiac reserve but also predispose to arrhythmogenesis. This is congruent with the arrhythmic potential of chronic stress in humans, replicated in animal models (Grippo et al. 2004; Liang et al. 2015). For example, social stress in rats increases arrhythmia vulnerability in association with gene changes suggestive of electromechanical remodeling and mitochondrial dysfunction (Andolina et al. 2021).

Similar mechanisms may participate in the stress‐dependent decline in inotropy and lusitropy in hearts devoid of external neurohumoral influences and beating at a fixed rate and degree of ventricular stretch (Figure 2). Zhang et al. (2019) observed both a reduction in contractility and increased arrhythmogenesis (associated with hypertrophic remodeling) in mice subjected to restraint stress, potentially involving epigenetic modulation of cardiomyopathy and adrenergic signaling processes. Chronic social stress in rats is also reported to impair cardiomyocyte contraction and relaxation in association with changes in Ca^2+^ dynamics and handling (including changes in sarcoplasmic reticulum Ca^2+^‐ATPase and phospholamban expression) and mitochondrial bioenergetics (Barbetti et al. 2022; Barbetti et al. 2023). Furthermore, depressive behavior in pregnant dams may not only increase heart rate and impair diastolic function, but also reduce lusitropy and contractility in offspring hearts (Czarzasta et al. 2021). Coupled with impaired cardiac resilience, such stress‐dependent changes may curtail cardiac efficiency and functional reserve, while increasing vulnerability to damage and disease.

#### 4.1.2 Intrinsic Resilience and “Adaptability”

Adaptations to stress not only involve top‐down or systems level control—allostatic resetting of physiological “steady states” (McEwen 1998) or shifts in ranges of reactive scope (Romero et al. 2009) or adaptive homeostasis (Davies 2016)—but also localized adaptations in tissue resilience via preconditioning or hormesis. These two adaptive processes enhance resilience in response to non‐damaging or damaging stressors, respectively. We have documented impaired intrinsic and adaptive resilience (PC) with aging and diabetes (Ashton et al. 2023; Peart et al. 2014; Russell et al. 2019), both conditions linked to cumulative AL (Bobba‐Alves et al. 2023; Danese and McEwen 2012) and disrupted adaptive homeostasis (Pomatto and Davies 2017). However, relationships between different stressors, AL, and the efficacy of conditioning (or hormesis) responses have not been previously studied. We find PC is functional in CD and WD hearts, whereas CS significantly inhibits this adaptive protective response. An unexpected ability of the WD to partially counter this dysfunction suggests some metabolic involvement in this abnormality. Though not feasible to directly incorporate dysfunctional adaptation into overall measures of cardiac and systemic AL (as we are unable to assess both intrinsic and adaptive resilience in a single heart), the differing resilience's and ALs for non‐PC and PC hearts identify additional CS‐dependent impacts on cardiovascular vulnerability.

### 4.2 Proteome Changes Implicate Mitochondrial and Innate Defense Processes

As in prior analysis of cardiac aging (Ashton et al. 2023; Peart et al. 2014), stress‐ and diet‐dependent reductions in cardiac resilience and adaptation were associated with shifts in mitochondrial function and innate defense pathways. The prominence of mitochondrial processes is congruent with the energetic model of allostasis, in which the energy costs of chronic allostasis impinge on reserves available for growth and repair processes (Bobba‐Alves et al. 2022). In humans, such imbalance may be worsened by the anticipatory nature of allostasis: increased energy expenditure without a survival “payoff” representing a futile cost. Stress can augment energy expenditure by 65% in humans, with glucocorticoids known to modify bioenergetics, mitochondrial biogenesis, and function (Bobba‐Alves et al. 2022; Du et al. 2009). We find that psychosocial and metabolic stressors (and PC) predominantly influence energy generation/mitochondrial pathways, together with innate defenses. Other studies are also consistent with the energetic model of allostasis, and a dominant role for mitochondria in diet and stress effects. For example, a maternal high‐fat diet induces metabolic stress and mitochondrial dysfunction in the hearts of offspring (Mdaki et al. 2016). In addition, while WD feeding may initially improve mitochondrial energy generation, chronic dietary stress results in mitochondrial dysfunction and cardiomyopathy (Mourmoura et al. 2016). This confirms a hyper‐metabolic state in response to metabolic stress and a biological cost in terms of disease risk.

Shifts in innate defenses (cell immunity, xenobiotic metabolism, and glutathione anti‐oxidant defense) are also consistent with aging‐related changes in mouse hearts (Ashton et al. 2023), shifts linked to impaired intrinsic and adaptive resilience (Peart et al. 2014). This broadly agrees with the notion that the influences of chronic stress reflect in part an acceleration of biological aging. For biological wear‐and‐tear to accumulate sufficiently to influence functionality and resilience/adaptation, intrinsic defenses and quality control and repair mechanisms must be overwhelmed/suppressed (and bioenergetic state impaired), as implicated and observed in models of aging (Ashton et al. 2023; Gems and McElwee 2005; Gladyshev 2016) and consistent with diet/stress dependent pathway changes here.

In terms of adaptive PC, mitochondrial/respiratory chain proteins and *Z*‐disk/sarcomeric proteins were the most significantly modified in CD hearts, with downregulation of mitochondrial and oxidative phosphorylation proteins and upregulation of estrogen receptor, glutathione‐mediated detoxification, glycolysis, and xenobiotic metabolism pathways. Adaptive protection may thus involve modification of mitochondrial function and innate defense pathways in response to prior stress. Marginal differences in PC‐sensitive pathway responses in WD and CD hearts are consistent with preserved efficacy of PC in both diet groups. Differences in PC proteome responses in (unprotected) CD + CS hearts versus (protected) CD hearts include a relative reduction in mitochondrial dysfunction proteins and an increase in oxidative phosphorylation, sirtuin, and RHODGI pathways. While warranting deeper interrogation, this also supports a key role for mitochondria in the efficacy of PC.

We must nonetheless exercise caution in interpreting proteome changes, as hearts were also subjected to I‐R stress before sampling and analysis. Thus, proteome outcomes may involve both direct cardiac effects of diet and stress, together with their influences on I‐R tolerance. This renders the question of causality problematic, since worsened I‐R tolerance with the WD or exaggerated inflammation with CS may influence proteome responses. Nonetheless, whether a result of direct effects and/or influences on I‐R tolerance, mitochondrial/energy metabolism, and innate defense pathways are among the most significantly modified by stress and WD feeding.

### 4.3 Individual and Comorbid Influences of Dietary and Psychosocial Stress on Allostatic Loads—Relevance to Multimorbidity

Allostasis and related models provide a framework for understanding interactions between chronic stressors (Danese and McEwen 2012; Korte et al. 2005) and how shifts in homeostatic ranges influence both disease development and therapeutic strategies. Seeman et al. (1997) first described an AL index over 25 years ago, summating binary scores for 10 neuroendocrine, metabolic, and cardiovascular biomarkers falling either within or outside the highest risk quartiles. Despite a diversity of biomarkers and mathematical approaches in subsequent work, clinical investigations broadly confirm consistent relationships between AL and both conventional and unconventional risk factors, with an improved predictive power of AL in terms of disease risk and mortality (Gillespie et al. 2019; Li et al. 2024; Parker et al. 2022; Steptoe et al. 2014). These and other studies (Gruenewald et al. 2006) confirm the importance of multiple biological systems and mechanistic pathways in determining disease risk and outcomes. Analysis of AL here demonstrates that individual subsystem and systemic loads respond to dietary and psychosocial stressors in distinct, even opposing ways; cardiovascular and behavioral subsystems are particularly sensitive to comorbid WD and CS; and initially beneficial influences of CS on metabolic and neuro‐endocrine‐immune AL do not necessarily translate to improved cardiovascular or behavioral outcomes and loads. Constituent subsystems within meta (systemic) AL measures have been previously shown to exhibit distinct profiles with differing stressors and to differ in the strength of their associations with disease or mortality. Others report differing contributions of individual elements to cognitive or physical outcomes (Karlamangla et al. 2002). The present findings not only confirm unique influences of diet and stress on subsystem outcomes and AL, but are consistent with co‐development of cardiovascular and affective disorders as a result of positive interactions between two widely shared environmental risks—psychosocial stress and a Western‐type diet.

#### 4.3.1 Stress and Diet Independently and Interactively Influence Subsystem Loads

Recent meta‐analysis confirms the importance of combined rather than individual lifestyle behaviors (including poor diet) in explaining associations between stressors, allostasis, and disease (Mattei et al. 2013; Zhang et al. 2023; Zhou et al. 2022). A small number of human studies have examined relationships between diet and allostatic loads (e.g., Mattei et al. 2013; Zhang et al. 2023; Zhou et al. 2022), supporting consistent relationships between AL and dietary factors linked to health, longevity, and disease risk. Importantly, incompletely defined interactions between stressors may be crucial in determining disease risk and outcomes. For example, Daubenmier et al. (2007) report that improvements in dietary fat and stress management (together with exercise) are “individually, additively and interactively related to coronary risk.” In analysis of the widowhood effect (mortality after loss of a long‐term spouse), Fagundes and Wu present evidence that the impacts of grief/negative emotion and poor diet on CVD and mortality are not simply additive but synergistic (Fagundes and Wu 2021). The authors highlight the need for further study of the poorly defined interactions between diet and grief or other forms of emotional stress.

Interestingly, we acquire evidence of both positive and negative interactions between the WD and CS, and distinct influences on subsystem or systemic AL. While systemic AL is not worsened by comorbid CS and a WD (due in part to counterbalancing influences on metabolic and neuro‐endocrine‐immune ALs), the cardiovascular impacts of diet and stress appear additive, with comorbidity inducing a broader array of cardiovascular dysfunctions and increasing AL. The behavioral effects of the two stressors are also unique, with anhedonia induced in WD mice and anxiogenesis with CS. Two key elements of MDD, these outcomes suggest anhedonia and anxiety may be mediated by independent biological mechanisms (rather than temporally distinct outcomes of a common mechanistic path). Collectively, these observations indicate comorbid dietary and psychosocial stressors increase the breadth of cardiovascular and behavioral pathologies and appear necessary to increase cardiovascular AL and induce a depressive phenotype.

Prior studies have identified diverse interactions between chronic stress and poor diet, including improved stress tolerance with high fat/sugar diets (Kanazawa et al. 2003; Maniam et al. 2016), and resistance to the metabolic effects of a high fat/sugar diet as a result of early life stress (Maniam et al. 2015). Other work indicates that dietary obesity worsens or prolongs the cardiovascular impacts of stress (Sedová et al. 2004). Our prior findings are mixed, with evidence for additive effects of adult or early life stress on the metabolic and behavioral effects of a WD (Robertson et al. 2024), or beneficial influences of stress on cardiometabolic risk factors in WD‐fed mice (Hatton‐Jones et al. 2022). However, and consistent with the current data, the metabolic influences of stress in these studies were not associated with changes in cardiac resilience. Despite differing outcomes across past studies, such work and the present findings collectively demonstrate that dietary and psychosocial stressors induce quite distinct, even opposing influences on different subsystems, and that improvements in one system (e.g., reductions in metabolic or neuro‐endocrine‐immune AL) do not necessarily benefit cardiovascular or behavioral systems and loads.

The metabolic influence of CS likely stems from significant weight loss (relative to unstressed animals). Chronic stress is most widely linked to weight loss in mammals, consistent with relative weight loss in people with “typical” MDD (Patist et al. 2018). This contrasts with weight gain in less prevalent atypical MDD, which is more prominent in younger age groups and females and may involve distinct changes in satiety mechanisms and affective or reward pathway influences of highly palatable foods (Patist et al. 2018). Stress‐dependent weight loss also contrasts with weight gain in chronic glucocorticoid excess/Cushing syndrome, reflecting in part the transience of glucocorticoid changes with chronic and repeated stress (recovering to baseline within days in rodents) (Bowers et al. 2008). Furthermore, tissue‐specific generation of glucocorticoids by 11β‐hydroxysteroid dehydrogenase type 1 may be more important than circulating levels in determining body weight change (Heaselgrave et al. 2024). As for influences on circulating glucocorticoids, the effects of chronic stress on 11β‐hydroxysteroid dehydrogenase type 1 expression/activity also appear distinct from those with chronic glucocorticoid excess (Jellinck et al. 1997).

### 4.4 Study Limitations

There are limitations in the present study. First, this integrative analysis was undertaken in males, and further studies are warranted to test for similar or distinct outcomes in females. We also do not directly incorporate HPA axis mediators (beyond noradrenaline levels) in AL calculations. However, it is now clear that glucocorticoid responses are not strictly indicative of stress nor globally elevated by different stressors (e.g., starvation). As already discussed, additional measures of hedonic behavior would also be of value since sucrose preference may be unduly influenced by dietary interventions. Finally, as noted above, since we assess proteome changes in postischemic tissue, we effectively assess combined influences of diet/stress on the baseline (pre‐ischemic) phenotype and I‐R resilience. Further work in normoxic/nonischemic tissues would be of value.

### 4.5 Perspectives and Significance

The present investigation indicates that the cardiovascular and also behavioral subsystems are particularly sensitive to comorbid stress and a WD. While these stressors are not broadly additive in terms of systemic AL, there is evidence of additivity specifically within these two subsystems: each form of stress induces unique effects on the heart and behavior, and comorbid CS and a WD are necessary to both increase cardiovascular AL and induce a depressive phenotype. These observations support the co‐development of cardiovascular and affective disorders in response to common and interacting environmental stressors. Disturbances in mitochondrial energy metabolism and innate defenses—mirroring changes predicted with/underpinning biological aging—may participate in these diet and stress effects.

## Author Contributions

E.F.T., J.N.P., J.P.H., and T.H. conceived and designed the study; B.L., C.K., E.F.T., J.T.I., J.N.P., J.P.H., M.N., S.N., T.H., and T.Y. performed experiments and molecular analyses; E.F.T., J.N.P., J.P.H., M.N., N.J.C.S., S.N., and T.H. interpreted experimental data; E.F.T., J.N.P., J.P.H., M.N., and T.H. prepared figures and drafted the manuscript; B.L., C.K., E.F.T., J.T.I., J.N.P., J.P.H., M.N., N.J.C.S., S.N., T.H., and T.Y. edited, revised, and approved the final manuscript.

## Conflicts of Interest

The authors declare no conflicts of interest.

## Supporting information


**Data S1:** Supporting Information.

**Table S1:** List of the left ventricular proteins identified in the ventricles of the CD, WD, CD + CS, and WD + CS study groups.

## Data Availability

The data that support the findings of this study are available from the corresponding author upon reasonable request.

## References

[cph470045-bib-0001] Ajoolabady, A. , D. Pratico , D. Tang , S. Zhou , C. Franceschi , and J. Ren . 2024. “Immunosenescence and Inflammaging: Mechanisms and Role in Diseases.” Ageing Research Reviews 101: 102540. 10.1016/j.arr.2024.102540. 39395575

[cph470045-bib-0002] Andolina, D. , M. Savi , D. Ielpo , et al. 2021. “Elevated miR‐34a Expression and Altered Transcriptional Profile Are Associated With Adverse Electromechanical Remodeling in the Heart of Male Rats Exposed to Social Stress.” Stress (Amsterdam, Netherlands) 24, no. 5: 621–634. 10.1080/10253890.2021.1942830. 34227918

[cph470045-bib-0003] Ashton, K. J. , C. J. Kiessling , J. M. Thompson , et al. 2023. “Early Cardiac Aging Linked to Impaired Stress‐Resistance and Transcriptional Control of Stress Response, Quality Control and Mitochondrial Pathways.” Experimental Gerontology 171: 112011. 10.1016/j.exger.2022.112011. 36347360

[cph470045-bib-0004] Barbetti, M. , R. Vilella , C. Dallabona , et al. 2022. “Decline of Cardiomyocyte Contractile Performance and Bioenergetic Function in Socially Stressed Male Rats.” Heliyon 8, no. 11: e11466. 10.1016/j.heliyon.2022.e11466. 36387533 PMC9660606

[cph470045-bib-0005] Barbetti, M. , R. Vilella , V. Naponelli , et al. 2023. “Repeated Witness Social Stress Causes Cardiomyocyte Contractile Impairment and Intracellular Ca2+ Derangement in Female Rats.” Physiology & Behavior 271: 114339. 10.1016/j.physbeh.2023.114339. 37625474

[cph470045-bib-0006] Bobba‐Alves, N. , R. P. Juster , and M. Picard . 2022. “The Energetic Cost of Allostasis and Allostatic Load.” Psychoneuroendocrinology 146: 105951. 10.1016/j.psyneuen.2022.105951. 36302295 PMC10082134

[cph470045-bib-0007] Bobba‐Alves, N. , G. Sturm , J. Lin , et al. 2023. “Cellular Allostatic Load Is Linked to Increased Energy Expenditure and Accelerated Biological Aging.” Psychoneuroendocrinology 155: 106322. 10.1016/j.psyneuen.2023.106322. 37423094 PMC10528419

[cph470045-bib-0008] Booth, F. W. , S. E. Gordon , C. J. Carlson , and M. T. Hamilton . 2000. “Waging War on Modern Chronic Diseases: Primary Prevention Through Exercise Biology.” Journal of Applied Physiology 88, no. 2: 774–787. 10.1152/jappl.2000.88.2.774. 10658050

[cph470045-bib-0009] Booth, F. W. , and S. J. Lees . 2007. “Fundamental Questions About Genes, Inactivity, and Chronic Diseases.” Physiological Genomics 28, no. 2: 146–157. 10.1152/physiolgenomics.00174.2006. 17032813

[cph470045-bib-0010] Booth, F. W. , C. K. Roberts , and M. J. Laye . 2012. “Lack of Exercise is a Major Cause of Chronic Diseases.” Comprehensive Physiology 2, no. 2: 1143–1211. 10.1002/cphy.c110025. 23798298 PMC4241367

[cph470045-bib-0011] Bowers, S. L. , S. D. Bilbo , F. S. Dhabhar , and R. J. Nelson . 2008. “Stressor‐Specific Alterations in Corticosterone and Immune Responses in Mice.” Brain, Behavior, and Immunity 22, no. 1: 105–113. 10.1016/j.bbi.2007.07.012. 17890050 PMC2175078

[cph470045-bib-0012] Carnevali, L. , M. Trombini , S. Rossi , et al. 2013. “Structural and Electrical Myocardial Remodeling in a Rodent Model of Depression.” Psychosomatic Medicine 75, no. 1: 42–51. 10.1097/PSY.0b013e318276cb0d. 23257930

[cph470045-bib-0014] Costoli, T. , A. Bartolomucci , G. Graiani , D. Stilli , G. Laviola , and A. Sgoifo . 2004. “Effects of Chronic Psychosocial Stress on Cardiac Autonomic Responsiveness and Myocardial Structure in Mice.” American Journal of Physiology. Heart and Circulatory Physiology 286, no. 6: H2133–H2140. 10.1152/ajpheart.00869.2003. 14962836

[cph470045-bib-0015] Czarzasta, K. , M. Wojciechowska , A. Segiet‐Swiecicka , S. Borodzicz‐Jazdzyk , M. Niedziela , and E. M. Sajdel‐Sulkowska . 2021. “The Effect of Depressive‐Like Behavior in Pregnant Rat Dams on the Cardiovascular System in Their Offspring.” Stress 24, no. 5: 652–658. 10.1080/10253890.2020.1845646. 33222571

[cph470045-bib-0016] Danese, A. , and B. S. McEwen . 2012. “Adverse Childhood Experiences, Allostasis, Allostatic Load, and Age‐Related Disease.” Physiology & Behavior 106, no. 1: 29–39. 10.1016/j.physbeh.2011.08.019. 21888923

[cph470045-bib-0017] Daubenmier, J. J. , G. Weidner , M. D. Sumner , et al. 2007. “The Contribution of Changes in Diet, Exercise, and Stress Management to Changes in Coronary Risk in Women and Men in the Multisite Cardiac Lifestyle Intervention Program.” Annals of Behavioral Medicine 33, no. 1: 57–68. 10.1207/s15324796abm3301_7. 17291171

[cph470045-bib-0018] Davies, K. J. 2016. “Adaptive Homeostasis.” Molecular Aspects of Medicine 49: 1–7. 10.1016/j.mam.2016.04.007. 27112802 PMC4868097

[cph470045-bib-0019] Dibato, J. E. , O. Montvida , F. Zaccardi , et al. 2021. “Association of Cardiometabolic Multimorbidity and Depression With Cardiovascular Events in Early‐Onset Adult Type 2 Diabetes: A Multiethnic Study in the U.S.” Diabetes Care 44, no. 1: 231–239. 10.2337/dc20-2045. 33177170

[cph470045-bib-0020] Du, J. , Y. Wang , R. Hunter , et al. 2009. “Dynamic Regulation of Mitochondrial Function by Glucocorticoids.” Proceedings of the National Academy of Sciences of the United States of America 106, no. 9: 3543–3548. 10.1073/pnas.0812671106. 19202080 PMC2637276

[cph470045-bib-0021] Du Toit, E. F. , W. S. Tai , A. Cox , et al. 2020. “Synergistic Effects of Low‐Level Stress and a Western Diet on Metabolic Homeostasis, Mood, and Myocardial Ischemic Tolerance.” American Journal of Physiology. Regulatory, Integrative and Comparative Physiology 319, no. 3: R347–R357. 10.1152/ajpregu.00322.2019. 32755463

[cph470045-bib-0022] Duarte, J. O. , F. C. Cruz , R. M. Leão , C. S. Planeta , and C. C. Crestani . 2015. “Stress Vulnerability During Adolescence: Comparison of Chronic Stressors in Adolescent and Adult Rats.” Psychosomatic Medicine 77, no. 2: 186–199. 10.1097/PSY.0000000000000141. 25659080

[cph470045-bib-0023] Fagundes, C. P. , and E. L. Wu . 2021. “Biological Mechanisms Underlying Widowhood's Health Consequences: Does Diet Play a Role?” Comprehensive Psychoneuroendocrinology 7: 100058. 10.1016/j.cpnec.2021.100058. 35757059 PMC9216459

[cph470045-bib-0024] Finger, B. C. , T. G. Dinan , and J. F. Cryan . 2012. “The Temporal Impact of Chronic Intermittent Psychosocial Stress on High‐Fat Diet‐Induced Alterations in Body Weight.” Psychoneuroendocrinology 37, no. 6: 729–741. 10.1016/j.psyneuen.2011.06.015. 21783325

[cph470045-bib-0025] Fortin, M. , L. Lapointe , C. Hudon , A. Vanasse , A. L. Ntetu , and D. Maltais . 2004. “Multimorbidity and Quality of Life in Primary Care: A Systematic Review.” Health and Quality of Life Outcomes 2: 51. 10.1186/1477-7525-2-51. 15380021 PMC526383

[cph470045-bib-0026] Gems, D. , and J. J. McElwee . 2005. “Broad Spectrum Detoxification: The Major Longevity Assurance Process Regulated by Insulin/IGF‐1 Signaling?” Mechanisms of Ageing and Development 126, no. 3: 381–387. 10.1016/j.mad.2004.09.001. 15664624

[cph470045-bib-0027] Gillespie, S. L. , C. M. Anderson , S. Zhao , et al. 2019. “Allostatic Load in the Association of Depressive Symptoms With Incident Coronary Heart Disease: The Jackson Heart Study.” Psychoneuroendocrinology 109: 104369. 10.1016/j.psyneuen.2019.06.020. 31307010 PMC7232849

[cph470045-bib-0028] Gladyshev, V. N. 2016. “Aging: Progressive Decline in Fitness due to the Rising Deleteriome Adjusted by Genetic, Environmental, and Stochastic Processes.” Aging Cell 15, no. 4: 594–602. 10.1111/acel.12480. 27060562 PMC4933668

[cph470045-bib-0029] Gould, T. D. , D. T. Dao , and C. E. Kovacsics . 2009. “Mood and Anxiety Related Phenotypes in Mice: Characterization Using Behavioral Tests.” In The Open Field Test, edited by T. D. Gould , 1–20. Humana Press.

[cph470045-bib-0030] Grippo, A. J. , J. A. Moffitt , M. K. Henry , et al. 2015. “Altered Connexin 43 and Connexin 45 Protein Expression in the Heart as a Function of Social and Environmental Stress in the Prairie Vole.” Stress (Amsterdam, Netherlands) 18, no. 1: 107–114. 10.3109/10253890.2014.979785. 25338193 PMC4675659

[cph470045-bib-0031] Grippo, A. J. , J. A. Moffitt , and A. K. Johnson . 2002. “Cardiovascular Alterations and Autonomic Imbalance in an Experimental Model of Depression.” American Journal of Physiology. Regulatory, Integrative and Comparative Physiology 282, no. 5: R1333–R1341. 10.1152/ajpregu.00614.2001. 11959673

[cph470045-bib-0032] Grippo, A. J. , C. M. Santos , R. F. Johnson , et al. 2004. “Increased Susceptibility to Ventricular Arrhythmias in a Rodent Model of Experimental Depression.” American Journal of Physiology. Heart and Circulatory Physiology 286, no. 2: H619–H626. 10.1152/ajpheart.00450.2003. 14715499

[cph470045-bib-0033] Gruenewald, T. L. , T. E. Seeman , C. D. Ryff , A. S. Karlamangla , and B. H. Singer . 2006. “Combinations of Biomarkers Predictive of Later Life Mortality.” Proceedings of the National Academy of Sciences of the United States of America 103, no. 38: 14158–14163. 10.1073/pnas.0606215103. 16983099 PMC1599928

[cph470045-bib-0034] Guthrie, B. , K. Payne , P. Alderson , M. E. McMurdo , and S. W. Mercer . 2012. “Adapting Clinical Guidelines to Take Account of Multimorbidity.” BMJ (Clinical Research Ed) 345: e6341. 10.1136/bmj.e6341. 23036829

[cph470045-bib-0035] Hare, D. L. , S. R. Toukhsati , P. Johansson , and T. Jaarsma . 2014. “Depression and Cardiovascular Disease: A Clinical Review.” European Heart Journal 35, no. 21: 1365–1372. 10.1093/eurheartj/eht462. 24282187

[cph470045-bib-0036] Hatton‐Jones, K. , A. J. Cox , J. N. Peart , J. P. Headrick , and E. F. du Toit . 2022. “Stress‐Induced Body Weight Loss and Improvements in Cardiometabolic Risk Factors Do Not Translate to Improved Myocardial Ischemic Tolerance in Western Diet‐Fed Mice.” Physiological Reports 10, no. 2: e15170. 10.14814/phy2.15170. 35076176 PMC8787728

[cph470045-bib-0037] Headrick, J. P. , J. Peart , B. Hack , A. Flood , and G. P. Matherne . 2001. “Functional Properties and Responses to Ischaemia‐Reperfusion in Langendorff Perfused Mouse Heart.” Experimental Physiology 86, no. 6: 703–716. 10.1111/j.1469-445x.2001.tb00035.x. 11698964

[cph470045-bib-0038] Heaselgrave, S. R. , S. Heising , S. A. Morgan , et al. 2024. “Glucocorticoid Excess Alters Metabolic Rate and Substrate Utilisation via 11β‐HSD1.” Journal of Endocrinology 263, no. 2: e240205. 10.1530/JOE-24-0205. 39302000 PMC11558800

[cph470045-bib-0039] Helman, T. J. , J. P. Headrick , J. N. Peart , and N. J. C. Stapelberg . 2022. “Central and Cardiac Stress Resilience Consistently Linked to Integrated Immuno‐Neuroendocrine Responses Across Stress Models in Male Mice.” European Journal of Neuroscience 56, no. 4: 4333–4362. 10.1111/ejn.15747. 35763309

[cph470045-bib-0040] Helman, T. J. , J. P. Headrick , J. Vider , J. N. Peart , and N. J. C. Stapelberg . 2022. “Sex‐Specific Behavioral, Neurobiological, and Cardiovascular Responses to Chronic Social Stress in Mice.” Journal of Neuroscience Research 100, no. 11: 2004–2027. 10.1002/jnr.25115. 36059192

[cph470045-bib-0041] Hu, F. B. , J. E. Manson , M. J. Stampfer , et al. 2001. “Diet, Lifestyle, and the Risk of Type 2 Diabetes Mellitus in Women.” New England Journal of Medicine 345, no. 11: 790–797. 10.1056/NEJMoa010492. 11556298

[cph470045-bib-0042] Hughes, L. D. , M. E. McMurdo , and B. Guthrie . 2013. “Guidelines for People Not for Diseases: The Challenges of Applying UK Clinical Guidelines to People With Multimorbidity.” Age and Ageing 42, no. 1: 62–69. 10.1093/ageing/afs100. 22910303

[cph470045-bib-0043] Ioakeim‐Skoufa, I. , K. Atkins , and M. Á. Hernández‐Rodríguez . 2025. “Optimizing Real‐World Evidence Studies for Regulatory Decision‐Making and Impact Assessment in Pharmacovigilance.” British Journal of Clinical Pharmacology 91: 1092–1095. 10.1111/bcp.16393. 39821103

[cph470045-bib-0044] Jellinck, P. H. , F. S. Dhabhar , R. R. Sakai , and B. S. McEwen . 1997. “Long‐Term Corticosteroid Treatment but Not Chronic Stress Affects 11beta‐Hydroxysteroid Dehydrogenase Type I Activity in Rat Brain and Peripheral Tissues.” Journal of Steroid Biochemistry and Molecular Biology 60, no. 5–6: 319–323. 10.1016/s0960-0760(96)00197-5. 9219923

[cph470045-bib-0045] Kai, K. , I. Morimoto , E. Morita , et al. 2000. “Environmental Stress Modifies Glycemic Control and Diabetes Onset in Type 2 Diabetes Prone Otsuka Long Evans Tokushima Fatty (OLETF) Rats.” Physiology & Behavior 68, no. 4: 445–452. 10.1016/s0031-9384(99)00187-0. 10713283

[cph470045-bib-0046] Kanazawa, M. , C. Y. Xue , H. Kageyama , et al. 2003. “Effects of a High‐Sucrose Diet on Body Weight, Plasma Triglycerides, and Stress Tolerance.” Nutrition Reviews 61, no. 5 Pt 2: S27–S33. 10.1301/nr.2003.may.S27-S33. 12828189

[cph470045-bib-0047] Karlamangla, A. S. , B. H. Singer , B. S. McEwen , J. W. Rowe , and T. E. Seeman . 2002. “Allostatic Load as a Predictor of Functional Decline. MacArthur Studies of Successful Aging.” Journal of Clinical Epidemiology 55, no. 7: 696–710. 10.1016/s0895-4356(02)00399-2. 12160918

[cph470045-bib-0048] Kendall, K. M. , E. Van Assche , T. F. M. Andlauer , et al. 2021. “The Genetic Basis of Major Depression.” Psychological Medicine 51, no. 13: 2217–2230. 10.1017/S0033291721000441. 33682643

[cph470045-bib-0049] Korte, S. M. , J. M. Koolhaas , J. C. Wingfield , and B. S. McEwen . 2005. “The Darwinian Concept of Stress: Benefits of Allostasis and Costs of Allostatic Load and the Trade‐Offs in Health and Disease.” Neuroscience and Biobehavioral Reviews 29, no. 1: 3–38. 10.1016/j.neubiorev.2004.08.009. 15652252

[cph470045-bib-0050] Kuan, V. , S. Denaxas , P. Patalay , et al. 2023. “Identifying and Visualising Multimorbidity and Comorbidity Patterns in Patients in the English National Health Service: A Population‐Based Study.” Lancet Digital Health 5, no. 1: e16–e27. 10.1016/S2589-7500(22)00187-X. 36460578

[cph470045-bib-0051] Lehnert, T. , D. Heider , H. Leicht , et al. 2011. “Review: Health Care Utilization and Costs of Elderly Persons With Multiple Chronic Conditions.” Medical Care Research and Review 68, no. 4: 387–420. 10.1177/1077558711399580. 21813576

[cph470045-bib-0052] Li, Y. , C. Chen , Y. Wen , et al. 2024. “Impact of Baseline and Longitudinal Allostatic Load Changes on Incident Cardiovascular Disease and All‐Cause Mortality: A 7‐Year Population‐Based Cohort Study in China.” Journal of Affective Disorders 355: 487–494. 10.1016/j.jad.2024.03.124. 38548202

[cph470045-bib-0053] Liang, J. , X. Yuan , S. Shi , et al. 2015. “Effect and Mechanism of Fluoxetine on Electrophysiology in Vivo in a Rat Model of Postmyocardial Infarction Depression.” Drug Design, Development and Therapy 9: 763–772. 10.2147/DDDT.S75863. 25709400 PMC4330040

[cph470045-bib-0013] Major Depressive Disorder Working Group of the Psychiatric Genomics Consortium. Major Depressive Disorder Working Group of the Psychiatric Genomics Consortium . 2025. “Trans‐Ancestry Genome‐Wide Study of Depression Identifies 697 Associations Implicating Cell Types and Pharmacotherapies.” Cell 188, no. 3: 640–652.e9. 10.1016/j.cell.2024.12.002. 39814019 PMC11829167

[cph470045-bib-0054] Maniam, J. , C. P. Antoniadis , V. Le , and M. J. Morris . 2016. “A Diet High in Fat and Sugar Reverses Anxiety‐Like Behaviour Induced by Limited Nesting in Male Rats: Impacts on Hippocampal Markers.” Psychoneuroendocrinology 68: 202–209. 10.1016/j.psyneuen.2016.03.007. 26999723

[cph470045-bib-0055] Maniam, J. , C. P. Antoniadis , K. W. Wang , and M. J. Morris . 2015. “Early Life Stress Induced by Limited Nesting Material Produces Metabolic Resilience in Response to a High‐Fat and High‐Sugar Diet in Male Rats.” Frontiers in Endocrinology 6: 138. 10.3389/fendo.2015.00138. 26441828 PMC4561522

[cph470045-bib-0056] Marengoni, A. , S. Angleman , R. Melis , et al. 2011. “Aging With Multimorbidity: A Systematic Review of the Literature.” Ageing Research Reviews 10, no. 4: 430–439. 10.1016/j.arr.2011.03.003. 21402176

[cph470045-bib-0057] Mariani, N. , A. Borsini , C. A. M. Cecil , et al. 2021. “Identifying Causative Mechanisms Linking Early‐Life Stress to Psycho‐Cardio‐Metabolic Multi‐Morbidity: The Early Cause Project.” PLoS One 16, no. 1: e0245475. 10.1371/journal.pone.0245475. 33476328 PMC7819604

[cph470045-bib-0058] Mattei, J. , S. Bhupathiraju , and K. L. Tucker . 2013. “Higher Adherence to a Diet Score Based on American Heart Association Recommendations Is Associated With Lower Odds of Allostatic Load and Metabolic Syndrome in Puerto Rican Adults.” Journal of Nutrition 143, no. 11: 1753–1759. 10.3945/jn.113.180141. 24005611 PMC3796346

[cph470045-bib-0059] McEwen, B. S. 1998. “Stress, Adaptation, and Disease. Allostasis and Allostatic Load.” Annals of the New York Academy of Sciences 840: 33–44. 10.1111/j.1749-6632.1998.tb09546.x. 9629234

[cph470045-bib-0060] McEwen, B. S. , and J. C. Wingfield . 2003. “The Concept of Allostasis in Biology and Biomedicine.” Hormones and Behavior 43, no. 1: 2–15. 10.1016/s0018-506x(02)00024-7. 12614627

[cph470045-bib-0061] Mdaki, K. S. , T. D. Larsen , A. L. Wachal , et al. 2016. “Maternal High‐Fat Diet Impairs Cardiac Function in Offspring of Diabetic Pregnancy Through Metabolic Stress and Mitochondrial Dysfunction.” American Journal of Physiology. Heart and Circulatory Physiology 310, no. 6: H681–H692. 10.1152/ajpheart.00795.2015. 26801311 PMC4867345

[cph470045-bib-0062] Menotti, A. , I. Mulder , A. Nissinen , S. Giampaoli , E. J. Feskens , and D. Kromhout . 2001. “Prevalence of Morbidity and Multimorbidity in Elderly Male Populations and Their Impact on 10‐Year All‐Cause Mortality: The FINE Study (Finland, Italy, Netherlands, Elderly).” Journal of Clinical Epidemiology 54, no. 7: 680–686. 10.1016/s0895-4356(00)00368-1. 11438408

[cph470045-bib-0063] Mercanoglu, G. , N. Safran , H. Uzun , and L. Eroglu . 2008. “Chronic Emotional Stress Exposure Increases Infarct Size in Rats: The Role of Oxidative and Nitrosative Damage in Response to Sympathetic Hyperactivity.” Methods & Findings in Experimental & Clinical Pharmacology 30, no. 10: 745–752. 10.1358/mf.2008.30.10.1316822. 19271023

[cph470045-bib-0064] Mourmoura, E. , J. P. Rigaudière , K. Couturier , et al. 2016. “Long‐Term Abdominal Adiposity Activates Several Parameters of Cardiac Energy Function.” Journal of Physiology and Biochemistry 72, no. 3: 525–537. 10.1007/s13105-015-0427-7. 26255304

[cph470045-bib-0065] Nemati, M. , H. Zardooz , F. Rostamkhani , A. Abadi , and F. Foroughi . 2017. “High‐Fat Diet Effects on Metabolic Responses to Chronic Stress.” Archives of Physiology and Biochemistry 123, no. 3: 182–191. 10.1080/13813455.2017.1295083. 28276709

[cph470045-bib-0066] Parker, H. W. , A. M. Abreu , M. C. Sullivan , and M. K. Vadiveloo . 2022. “Allostatic Load and Mortality: A Systematic Review and Meta‐Analysis.” American Journal of Preventive Medicine 63, no. 1: 131–140. 10.1016/j.amepre.2022.02.003. 35393143

[cph470045-bib-0067] Patist, C. M. , N. J. C. Stapelberg , E. F. Du Toit , and J. P. Headrick . 2018. “The Brain‐Adipocyte‐Gut Network: Linking Obesity and Depression Subtypes.” Cognitive, Affective, & Behavioral Neuroscience 18, no. 6: 1121–1144. 10.3758/s13415-018-0626-0. 30112671

[cph470045-bib-0068] Peart, J. , and J. P. Headrick . 2003. “Adenosine‐Mediated Early Preconditioning in Mouse: Protective Signaling and Concentration Dependent Effects.” Cardiovascular Research 58, no. 3: 589–601. 10.1016/s0008-6363(03)00259-1. 12798432

[cph470045-bib-0069] Peart, J. N. , S. Pepe , M. E. Reichelt , et al. 2014. “Dysfunctional Survival‐Signaling and Stress‐Intolerance in Aged Murine and Human Myocardium.” Experimental Gerontology 50: 72–81. 10.1016/j.exger.2013.11.015. 24316036 PMC4096533

[cph470045-bib-0070] Peters, A. , and B. S. McEwen . 2012. “Introduction for the Allostatic Load Special Issue.” Physiology & Behavior 106, no. 1: 1–4. 10.1016/j.physbeh.2011.12.019. 22226993

[cph470045-bib-0071] Pomatto, L. C. D. , and K. J. A. Davies . 2017. “The Role of Declining Adaptive Homeostasis in Ageing.” Journal of Physiology 595, no. 24: 7275–7309. 10.1113/JP275072. 29028112 PMC5730851

[cph470045-bib-0072] Read, J. R. , L. Sharpe , M. Modini , and B. F. Dear . 2017. “Multimorbidity and Depression: A Systematic Review and Meta‐Analysis.” Journal of Affective Disorders 221: 36–46. 10.1016/j.jad.2017.06.009. 28628766

[cph470045-bib-0073] Reichelt, M. E. , L. Willems , B. A. Hack , J. N. Peart , and J. P. Headrick . 2009. “Cardiac and Coronary Function in the Langendorff‐Perfused Mouse Heart Model.” Experimental Physiology 94, no. 1: 54–70. 10.1113/expphysiol.2008.043554. 18723581

[cph470045-bib-0074] Robertson, K. , T. A. Griffith , T. J. Helman , et al. 2024. “Early Life Stress Exacerbates the Obesogenic and Anxiogenic Effects of a Western Diet Without Worsening Cardiac Ischaemic Tolerance in Male Mice.” Journal of Developmental Origins of Health and Disease 15: e14. 10.1017/S2040174424000205. 39291337

[cph470045-bib-0075] Romero, L. M. , M. J. Dickens , and N. E. Cyr . 2009. “The Reactive Scope Model ‐ a New Model Integrating Homeostasis, Allostasis, and Stress.” Hormones and Behavior 55, no. 3: 375–389. 10.1016/j.yhbeh.2008.12.009. 19470371

[cph470045-bib-0076] Rossi, A. , N. Mikail , S. Bengs , et al. 2022. “Heart‐Brain Interactions in Cardiac and Brain Diseases: Why Sex Matters.” European Heart Journal 43, no. 39: 3971–3980. 10.1093/eurheartj/ehac061. 35194633 PMC9794190

[cph470045-bib-0077] Royston, P. , D. G. Altman , and W. Sauerbrei . 2006. “Dichotomizing Continuous Predictors in Multiple Regression: A Bad Idea.” Statistics in Medicine 25, no. 1: 127–141. 10.1002/sim.2331. 16217841

[cph470045-bib-0078] Russell, J. S. , T. A. Griffith , T. Helman , E. F. Du Toit , J. N. Peart , and J. P. Headrick . 2019. “Chronic Type 2 but Not Type 1 Diabetes Impairs Myocardial Ischaemic Tolerance and Preconditioning in C57Bl/6 Mice.” Experimental Physiology 104, no. 12: 1868–1880. 10.1113/EP088024. 31535419

[cph470045-bib-0079] Russell, J. S. , T. A. Griffith , S. Naghipour , et al. 2020. “Dietary α‐Linolenic Acid Counters Cardioprotective Dysfunction in Diabetic Mice: Unconventional PUFA Protection.” Nutrients 12, no. 9: 2679. 10.3390/nu12092679. 32887376 PMC7551050

[cph470045-bib-0080] Ryan, A. , E. Wallace , P. O'Hara , and S. M. Smith . 2015. “Multimorbidity and Functional Decline in Community‐Dwelling Adults: A Systematic Review.” Health and Quality of Life Outcomes 13: 168. 10.1186/s12955-015-0355-9. 26467295 PMC4606907

[cph470045-bib-0081] Salis, Z. , B. Gallego , and A. Sainsbury . 2023. “Researchers in Rheumatology Should Avoid Categorization of Continuous Predictor Variables.” BMC Medical Research Methodology 23, no. 1: 104. 10.1186/s12874-023-01926-4. 37101144 PMC10134601

[cph470045-bib-0082] Sedová, L. , J. Bérubé , D. Gaudet , et al. 2004. “Diet‐Induced Obesity Delays Cardiovascular Recovery From Stress in Spontaneously Hypertensive Rats.” Obesity Research 12, no. 12: 1951–1958. 10.1038/oby.2004.245. 15687396

[cph470045-bib-0083] Seeman, T. E. , B. H. Singer , J. W. Rowe , R. I. Horwitz , and B. S. McEwen . 1997. “Price of Adaptation–Allostatic Load and Its Health Consequences. MacArthur Studies of Successful Aging.” Archives of Internal Medicine 157, no. 19: 2259–2268 Erratum in: Arch Intern Med 1999 Jun 14;159(11):1176. 9343003

[cph470045-bib-0085] Smith, S. M. , S. O'Kelly , and T. O'Dowd . 2010. “Gps' and Pharmacists' Experiences of Managing Multimorbidity: A ‘Pandora's Box’.” British Journal of General Practice 60, no. 576: 285–294. 10.3399/bjgp10X514756. PMC2894403 20594430

[cph470045-bib-0086] Stampfer, M. J. , F. B. Hu , J. E. Manson , E. B. Rimm , and W. C. Willett . 2000. “Primary Prevention of Coronary Heart Disease in Women Through Diet and Lifestyle.” New England Journal of Medicine 343, no. 1: 16–22. 10.1056/NEJM200007063430103. 10882764

[cph470045-bib-0087] Stapelberg, N. J. C. , D. L. Neumann , D. Shum , and J. P. Headrick . 2019. “Health, Pre‐Disease and Critical Transition to Disease in the Psycho‐Immune‐Neuroendocrine Network: Are There Distinct States in the Progression From Health to Major Depressive Disorder?” Physiology & Behavior 198: 108–119. 10.1016/j.physbeh.2018.10.014. 30393143

[cph470045-bib-0088] Steptoe, A. , R. A. Hackett , A. I. Lazzarino , et al. 2014. “Disruption of Multisystem Responses to Stress in Type 2 Diabetes: Investigating the Dynamics of Allostatic Load.” Proceedings of the National Academy of Sciences of the United States of America 111, no. 44: 15693–15698. 10.1073/pnas.1410401111. 25331894 PMC4226108

[cph470045-bib-0089] Taylor, J. M. G. , and M. Yu . 2002. “Bias and Efficiency Loss due to Categorizing an Explanatory Variable.” Journal of Multivariate Analysis 83: 248–263. 10.1006/jmva.2001.2045.

[cph470045-bib-0090] Townsend, A. , K. Hunt , and S. Wyke . 2003. “Managing Multiple Morbidity in Mid‐Life: A Qualitative Study of Attitudes to Drug Use.” BMJ (Clinical Research Ed) 327: 837. 10.1136/bmj.327.7419.837. PMC214019 14551097

[cph470045-bib-0091] Tran, J. , R. Norton , N. Conrad , et al. 2018. “Patterns and Temporal Trends of Comorbidity Among Adult Patients With Incident Cardiovascular Disease in the UK Between 2000 and 2014: A Population‐Based Cohort Study.” PLoS Medicine 15, no. 3: e1002513. 10.1371/journal.pmed.1002513. 29509757 PMC5839540

[cph470045-bib-0092] Tremblay, A. , and J. P. Chaput . 2012. “Obesity: The Allostatic Load of Weight Loss Dieting.” Physiology & Behavior 106, no. 1: 16–21. 10.1016/j.physbeh.2011.05.020. 21627975

[cph470045-bib-0093] Tyanova, S. , T. Temu , and J. Cox . 2016. “The MaxQuant Computational Platform for Mass Spectrometry‐Based Shotgun Proteomics.” Nature Protocols 11, no. 12: 2301–2319. 10.1038/nprot.2016.136. 27809316

[cph470045-bib-0094] Vancampfort, D. , C. U. Correll , B. Galling , et al. 2016. “Diabetes Mellitus in People With Schizophrenia, Bipolar Disorder and Major Depressive Disorder: A Systematic Review and Large Scale Meta‐Analysis.” World Psychiatry 15, no. 2: 166–174. 10.1002/wps.20309. 27265707 PMC4911762

[cph470045-bib-0095] Vogeli, C. , A. E. Shields , T. A. Lee , et al. 2007. “Multiple Chronic Conditions: Prevalence, Health Consequences, and Implications for Quality, Care Management, and Costs.” Journal of General Internal Medicine 22, no. Suppl 3: 391–395. 10.1007/s11606-007-0322-1. 18026807 PMC2150598

[cph470045-bib-0096] Zhang, P. , T. Li , Y. Q. Liu , et al. 2019. “Contribution of DNA Methylation in Chronic Stress‐Induced Cardiac Remodeling and Arrhythmias in Mice.” FASEB Journal 33, no. 11: 12240–12252. 10.1096/fj.201900100R. 31431066 PMC6902688

[cph470045-bib-0097] Zhang, S. , L. E , J. Pang , and X. Jiang . 2023. “Adults Allostatic Load Is Less With Greater Dietary Quality: National Health and Nutrition Examination Survey (NHANES) 2015–2018.” Asia Pacific Journal of Clinical Nutrition 32, no. 2: 227–235. 37382320 10.6133/apjcn.202306_32(2).0005

[cph470045-bib-0099] Zhao, Y. , J. Xu , J. Gong , and L. Qian . 2009. “L‐Type Calcium Channel Current Up‐Regulation by Chronic Stress Is Associated With Increased α1c Subunit Expression in Rat Ventricular Myocytes.” Cell Stress & Chaperones 14, no. 1: 33–41. 10.1007/s12192-008-0052-2. 18566917 PMC2673898

[cph470045-bib-0100] Zhou, M. S. , R. E. Hasson , A. Baylin , and C. W. Leung . 2022. “Associations Between Diet Quality and Allostatic Load in US Adults: Findings From the National Health and Nutrition Examination Survey, 2015‐2018.” Journal of the Academy of Nutrition and Dietetics 122, no. 12: 2207–2217. 10.1016/j.jand.2022.05.001. 35533873

