# Supplementary material for: Individual and Comorbid Influences of Chronic Stress and a Western Diet on Allostatic Loads and Cardiac Resilience, Adaptation and Proteome Profiles in Male Mice
Source: Compr Physiol. 2025 Sep 8;15(5):e70045. doi: 10.1002/cph4.70045 (PMC12415509; doi:10.1002/cph4.70045)
Supplement: Supplementary file 1 — Data S1: Supporting Information. [file CPH4-15-e70045-s002.pdf]

Western Diet (WD) Formulation

Fresh WD chow was made every 10 days. Chow contained a mix of:
- 1.6 kg lab chow, ground to powder (Irradiated Rat and Mouse Cubes; Specialty Feeds, Glen Forrest, Western Australia)
- 250 g solidified edible animal fat/oil (Supafry; Goodman Fielder Consumer Foods, Sydney, Australia)
- 280 g refined white sugar (Coles Group, Victoria, Australia)
- 4 x 397 g cans sweetened condensed milk (Coles Group, Victoria, Australia)

The mixture was rolled into balls, fully wrapped in baking paper and stored in an airtight container at 4°C for up to 10 days.

Nutrient analysis confirmed a caloric breakdown of:
32% calories from fat; 57% calories from carbohydrate; and 11% calories protein.

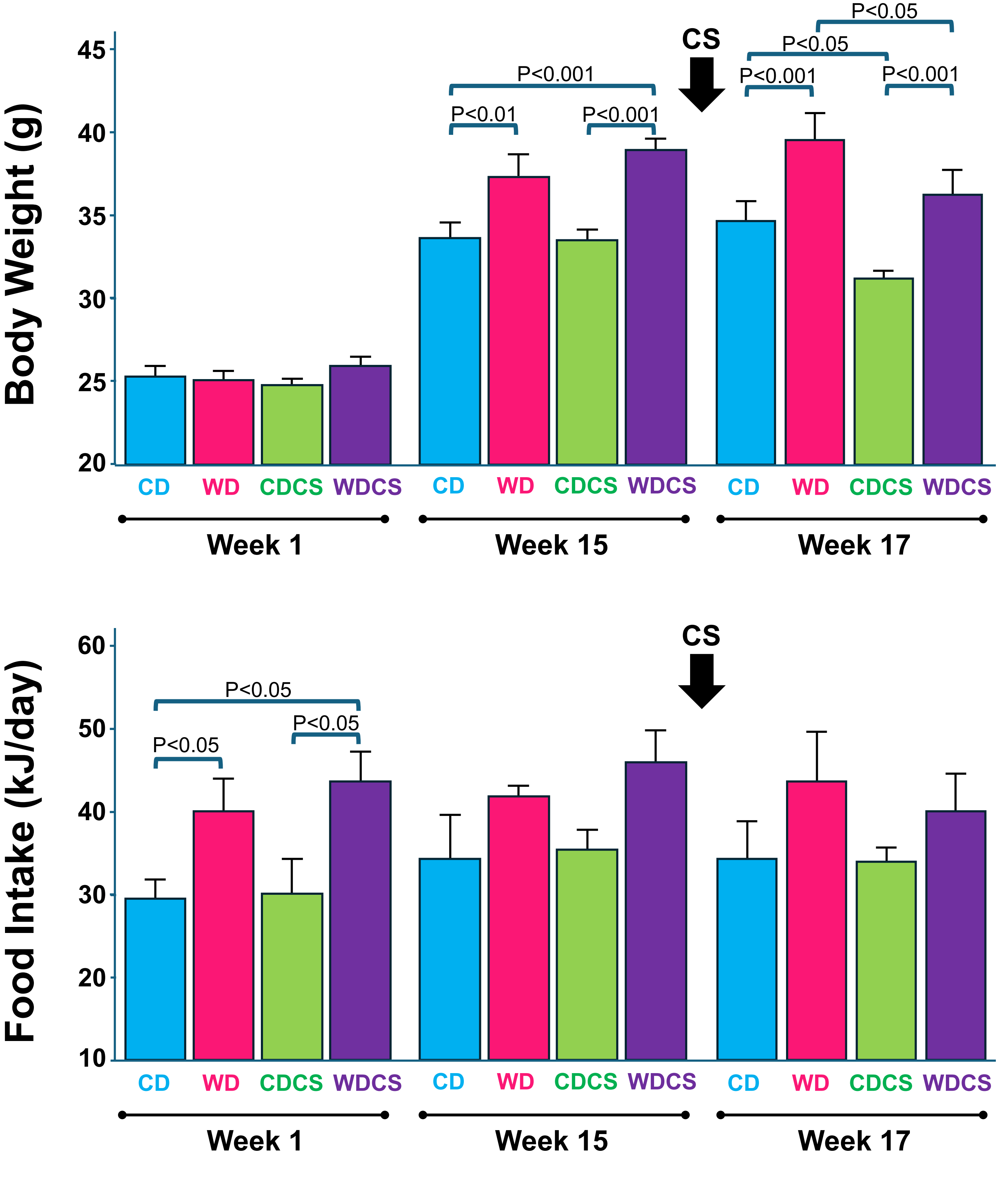

Fig. S1. Body weight changes and caloric intakes. Data shown for the 1st week of experimentation, week 15 (prior to induction of CS) and week 17 (end of experimentation)

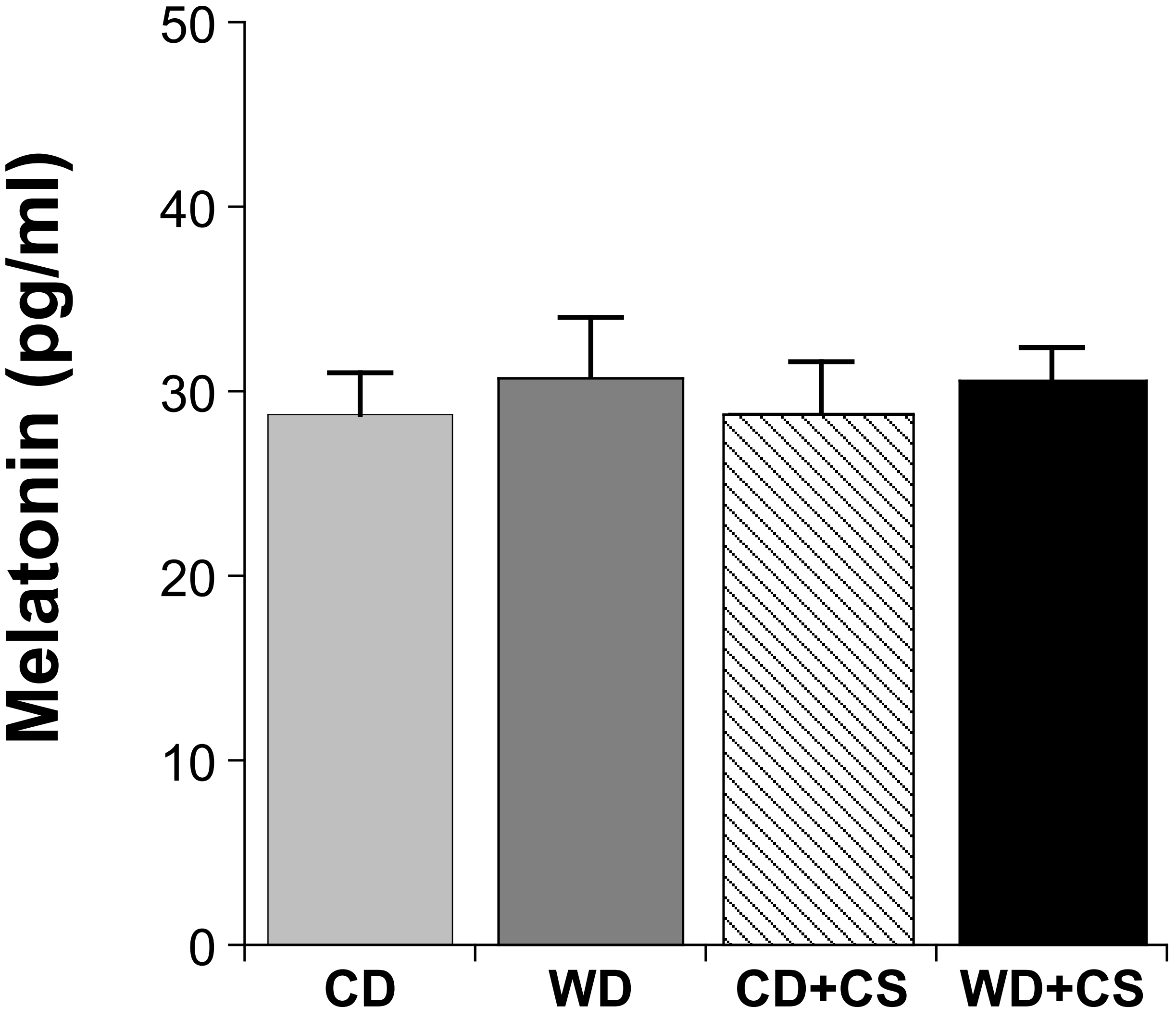

Fig. S2. Circulating melatonin levels. Melatonin was measured at the end of experimentation in mice fed a CD or WD for 17 wks without or with 2 hrs daily restraint stress over the final 2 wks (CD+CS, WD+CS). Data are means±SEM (n=8-13 per group). No differences in melatonin were detected between groups.

Fig. S3. Proteomic influence of stress in CD mice (CD+CS *vs.* CD). Shown are numbers of differentially expressed proteins, and the most modified Cellular Component, Molecular Function and Biological Processes.

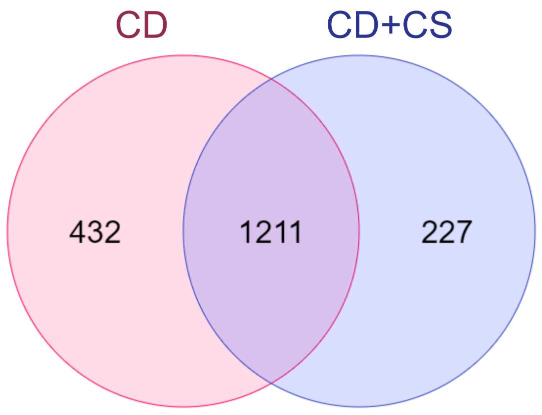

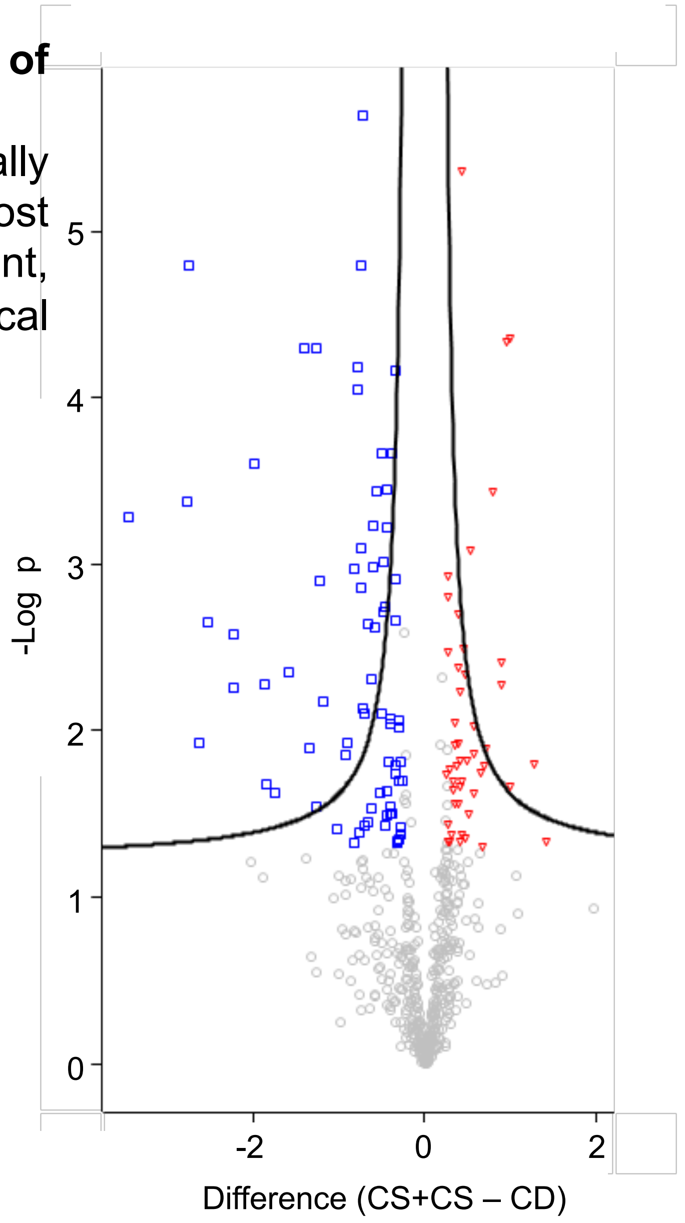

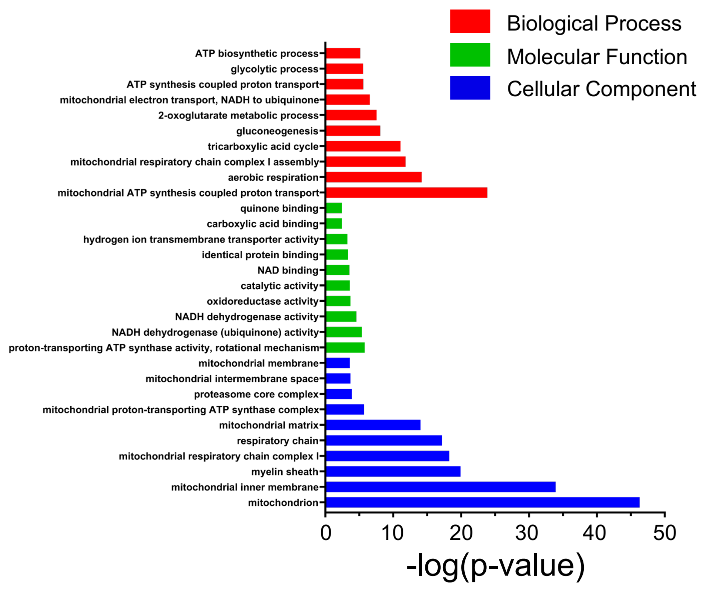

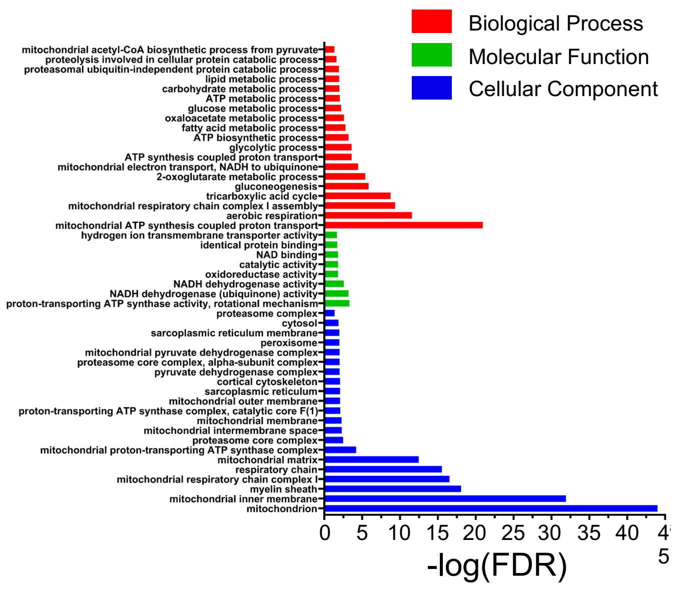

Fig. S4. Proteomic influence of WD feeding (WD *vs.* CD). Shown are numbers of differentially expressed proteins, and the most modified Cellular Component, Molecular Function and Biological Processes.

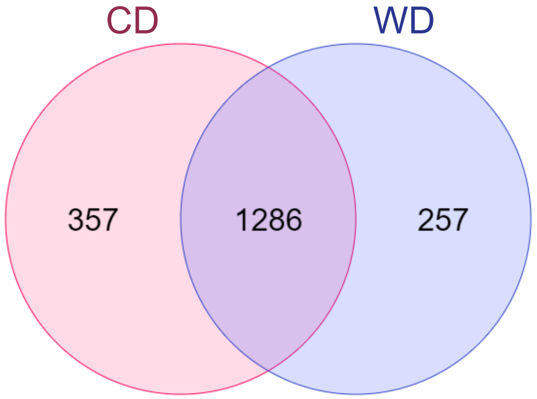

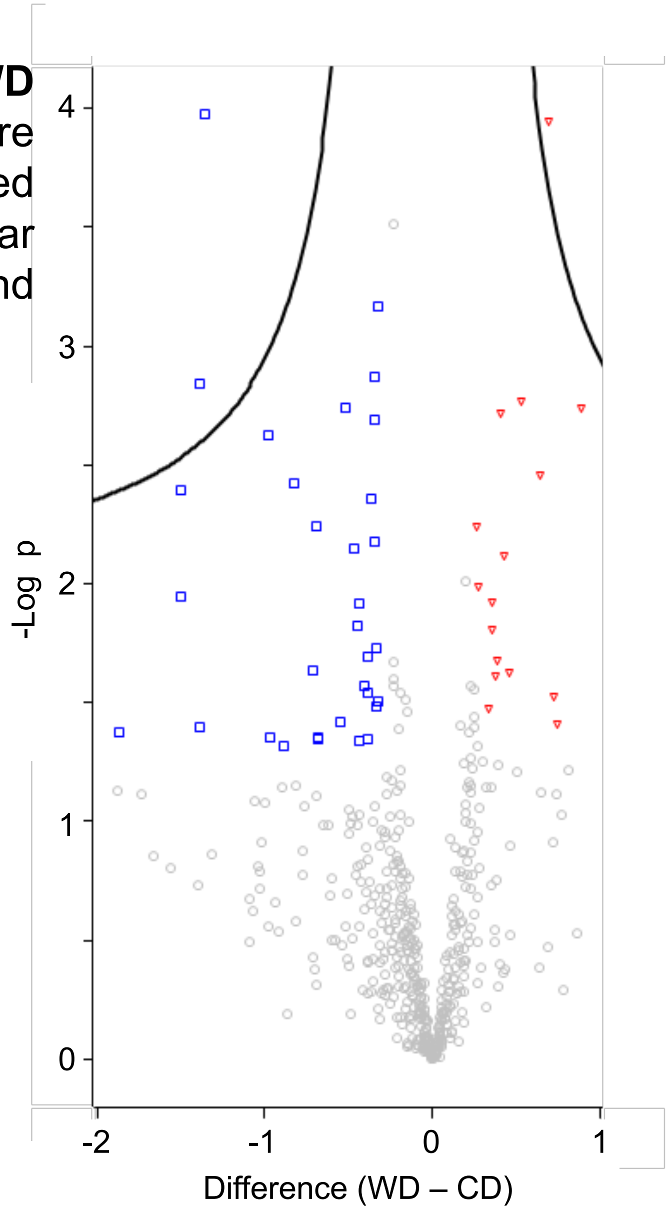

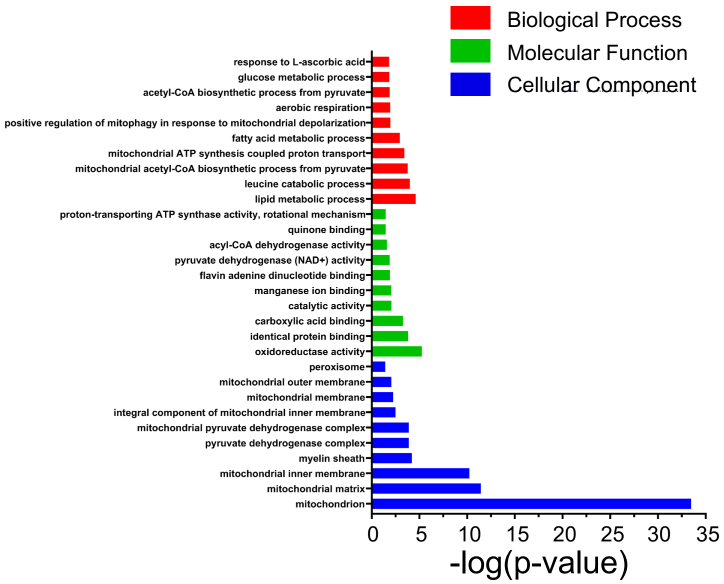

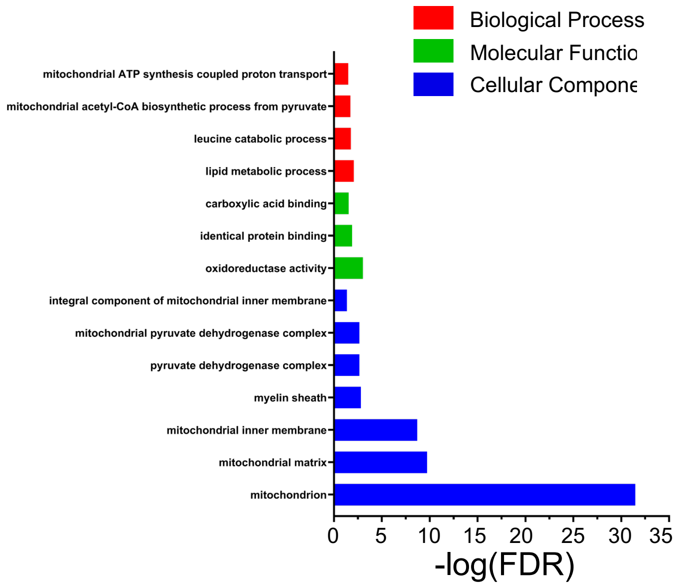

Fig. S5. Proteomic influence of stress in WD mice (WD+CS *vs.* WD). Shown are numbers of differentially expressed proteins, and the most modified Cellular Component, Molecular Function and Biological Processes.

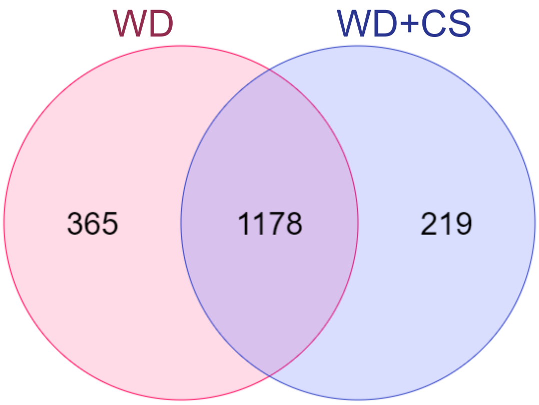

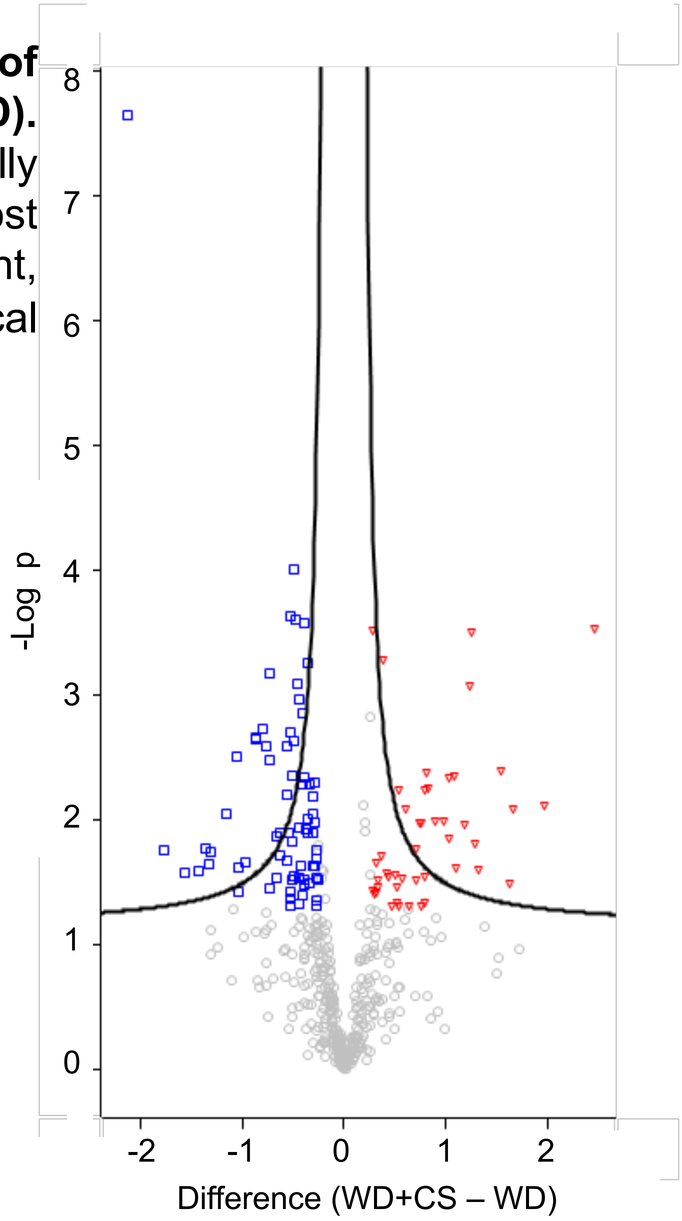

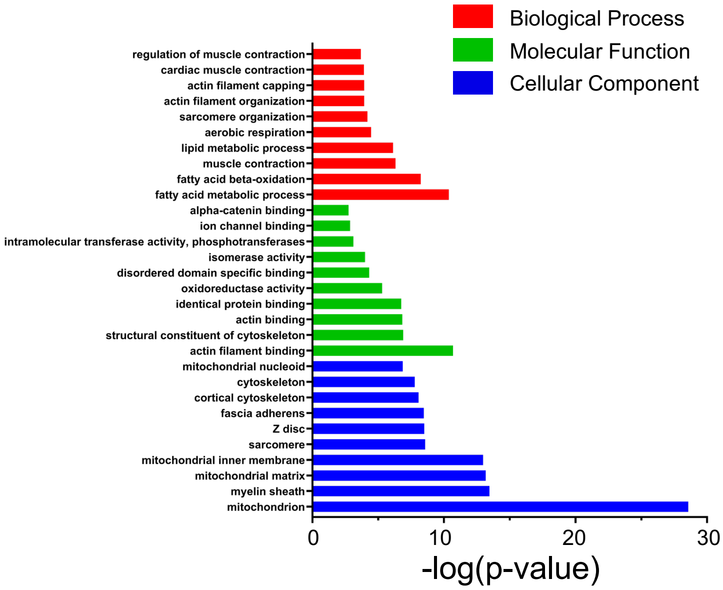

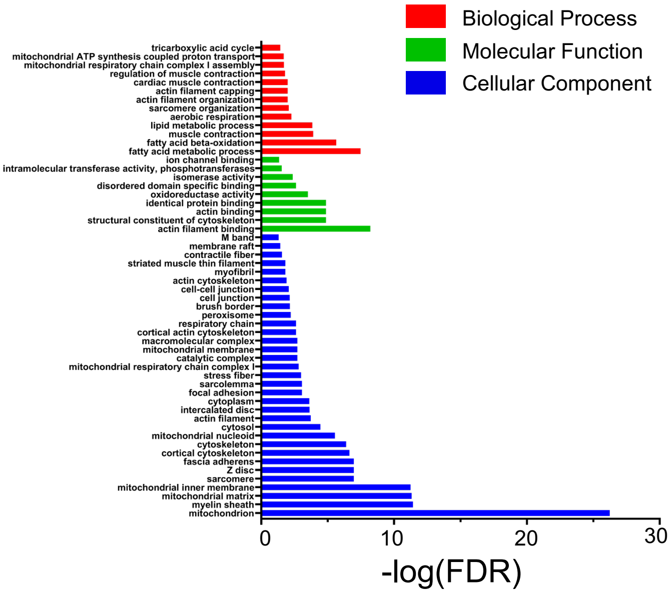

Fig. S6. Proteomic influence of the adaptive PC stimulus in CD mice (PC CD *vs*. CD). Shown are numbers of differentially expressed proteins, and the most modified Cellular Component, Molecular Function and Biological Process.

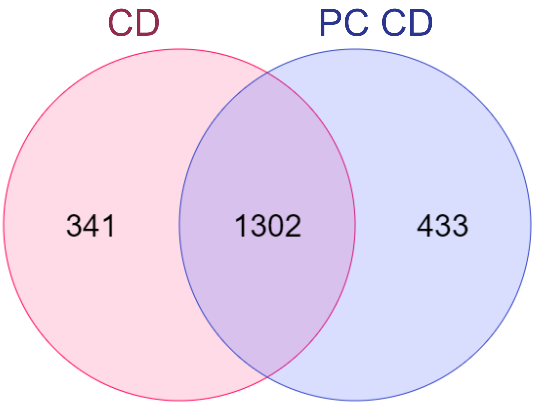

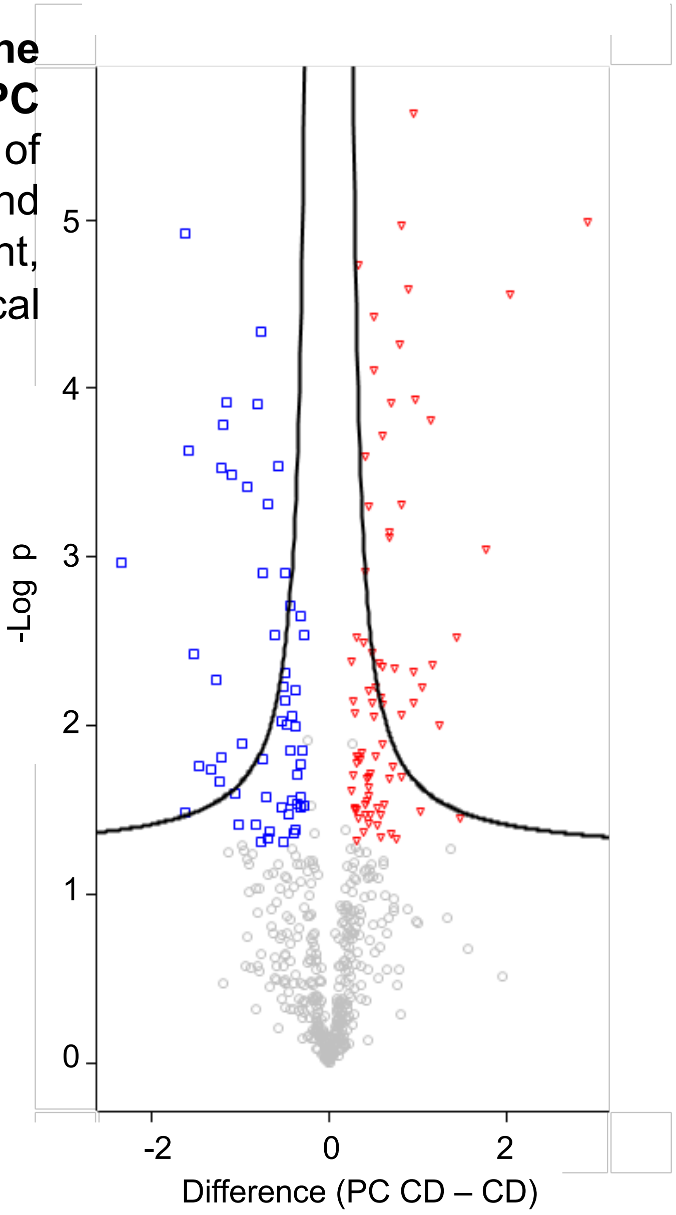

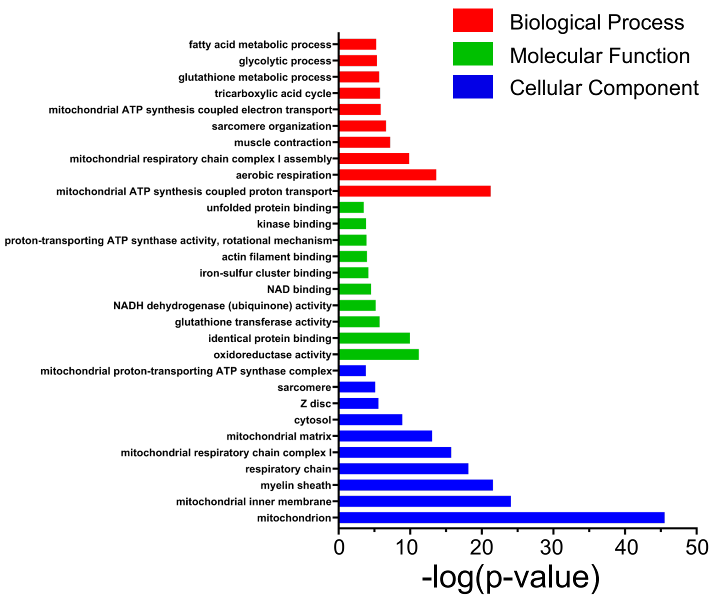

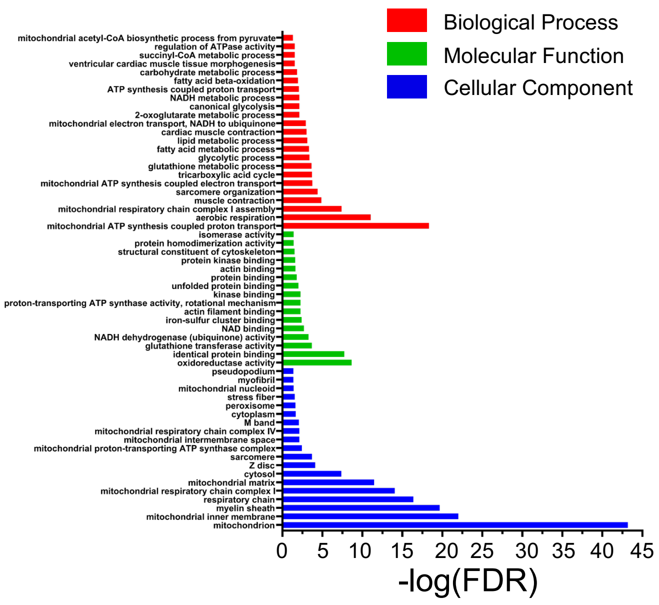

Fig. S7. Proteome influence of the adaptive PC stimulus in stressed CD mice (PC CD+CS *vs*. CD+CS). Shown are numbers of differentially expressed proteins, and the most modified Cellular Component, Molecular Function, Biological Processes.

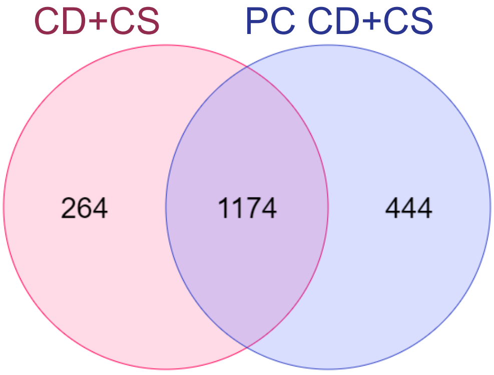

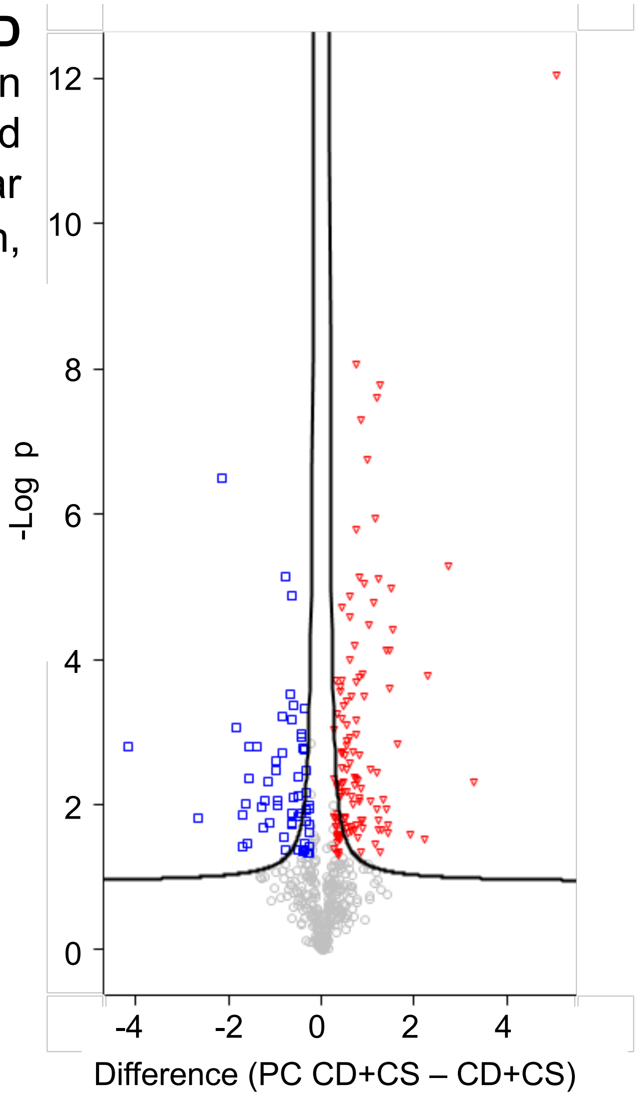

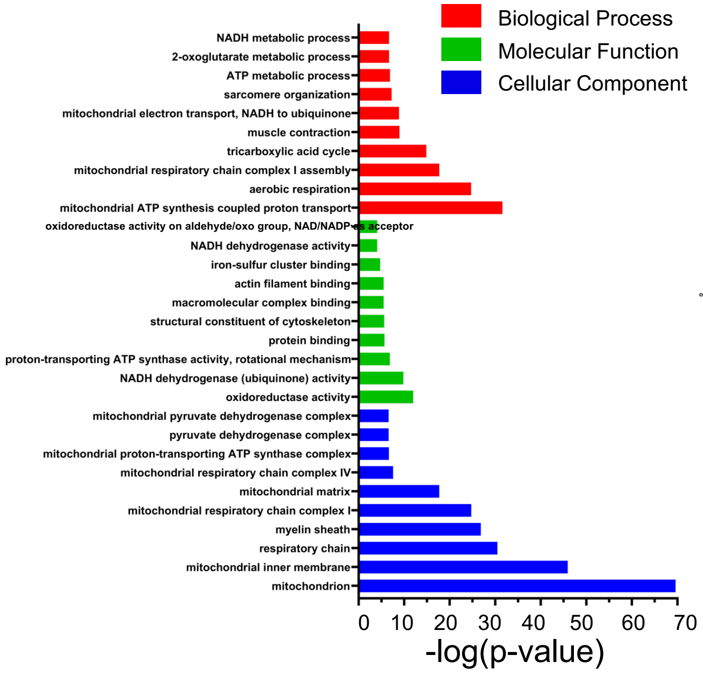

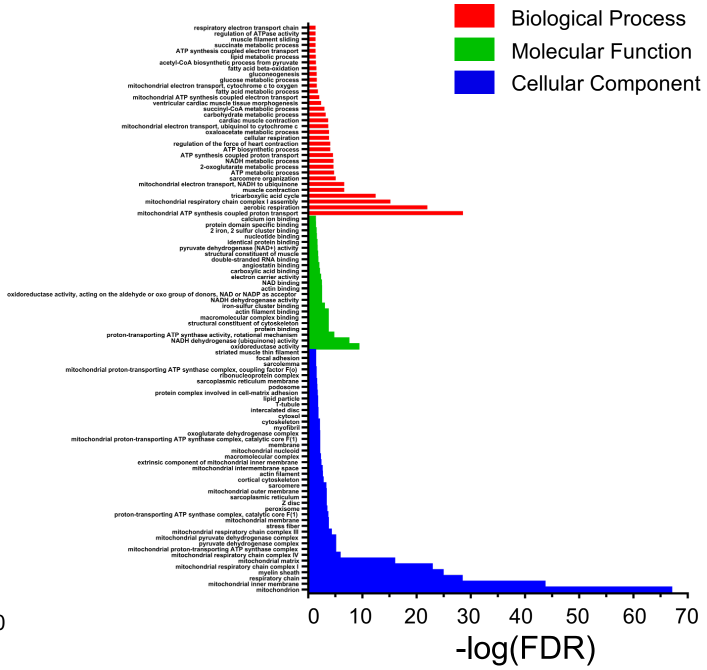

Fig. S8. Proteomic influence of the adaptive PC stimulus in WD mice (PC WD *vs*. WD). Shown are numbers of differentially expressed proteins, and the most modified Cellular Component, Molecular Function and Biological Process.

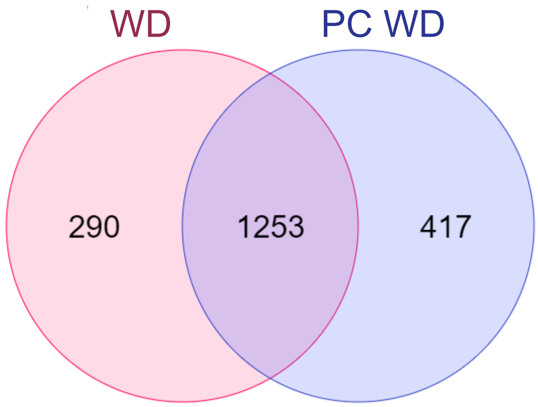

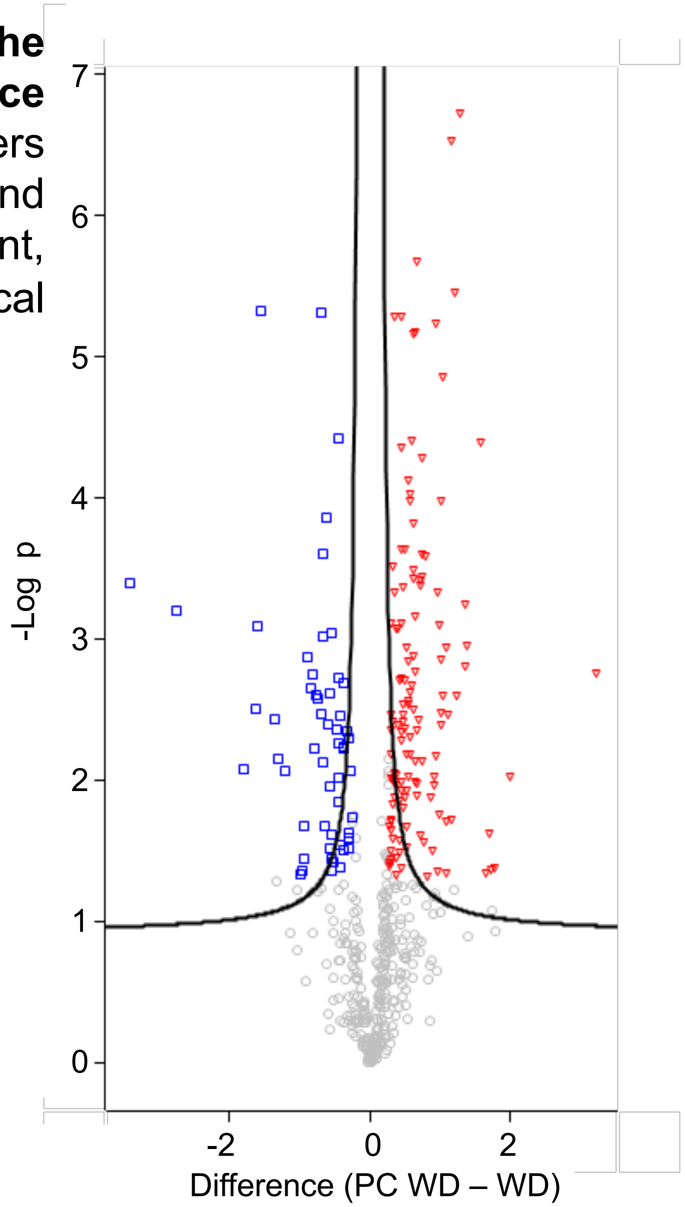

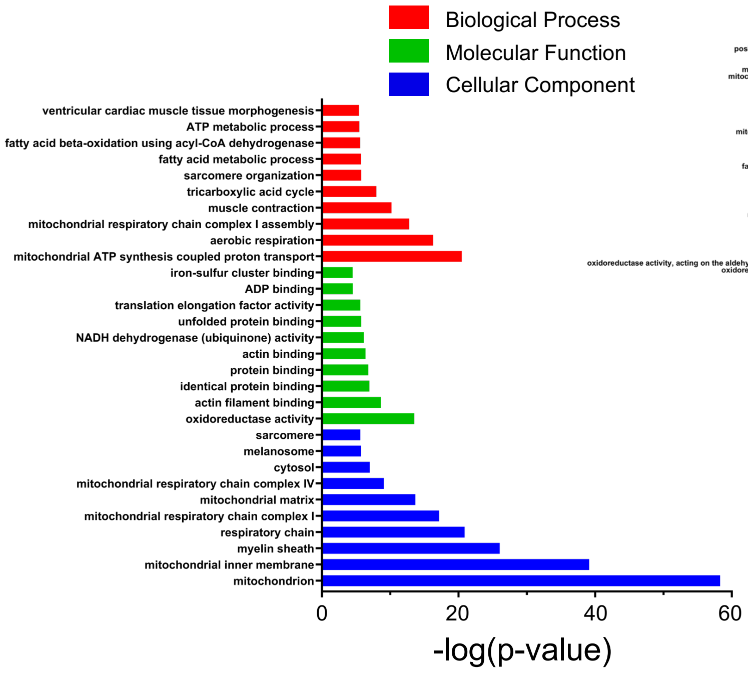

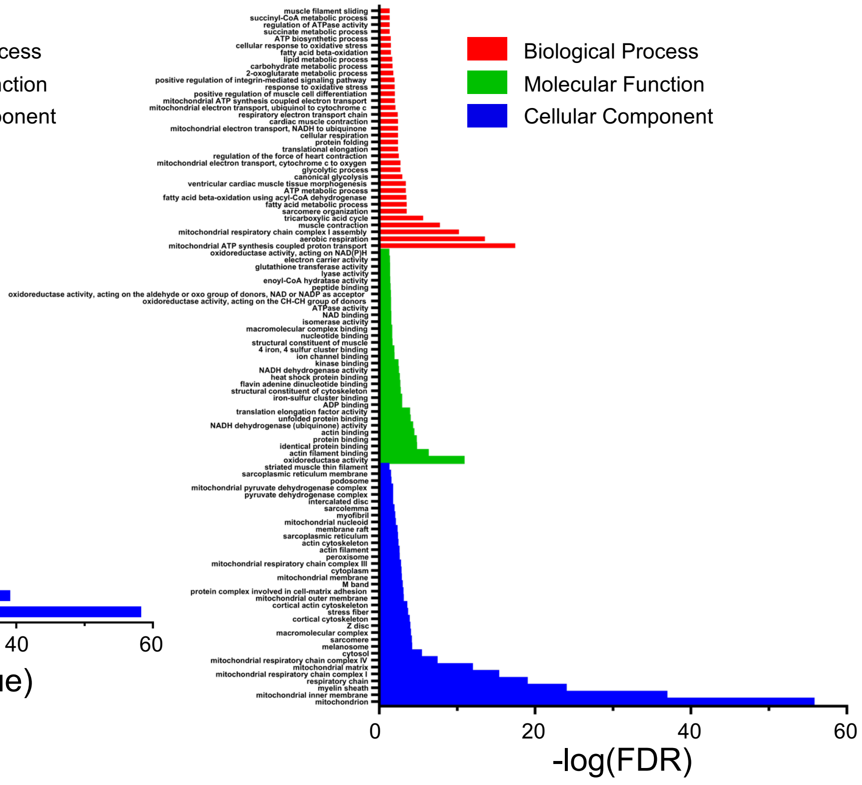

Fig. S9. Proteomic influence of the adaptive PC stimulus in stressed WD mice (PC WD+CS vs. WD+CS). Shown are numbers of differentially expressed proteins, Cellular Component, Molecular Function and Biological Processes.

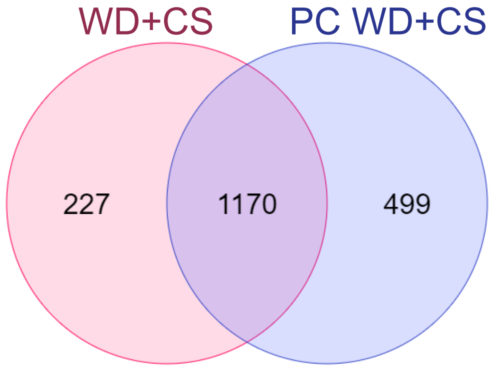

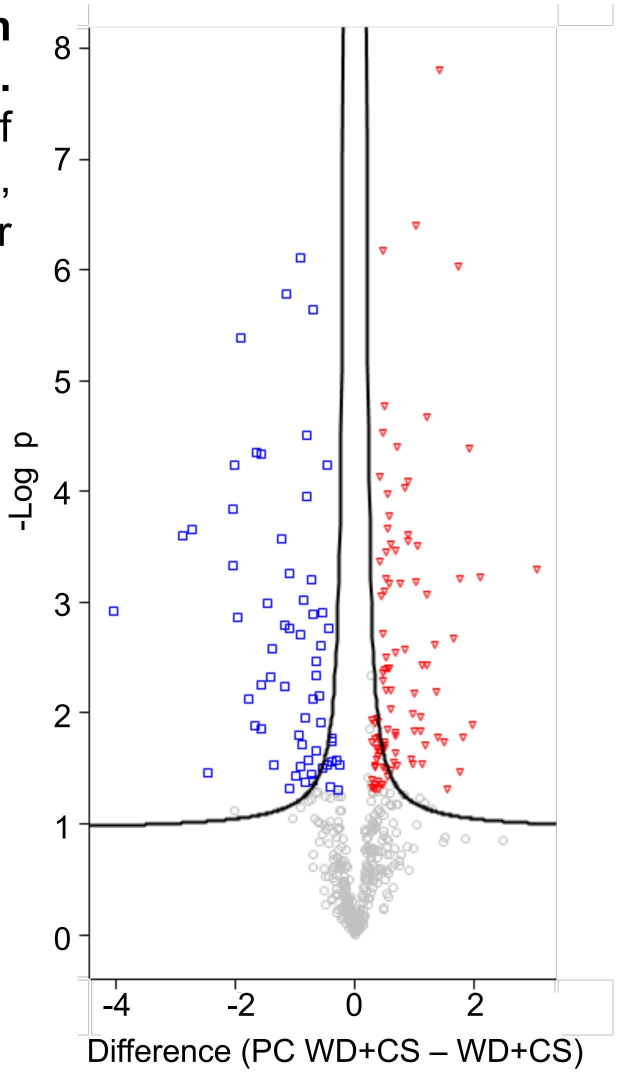

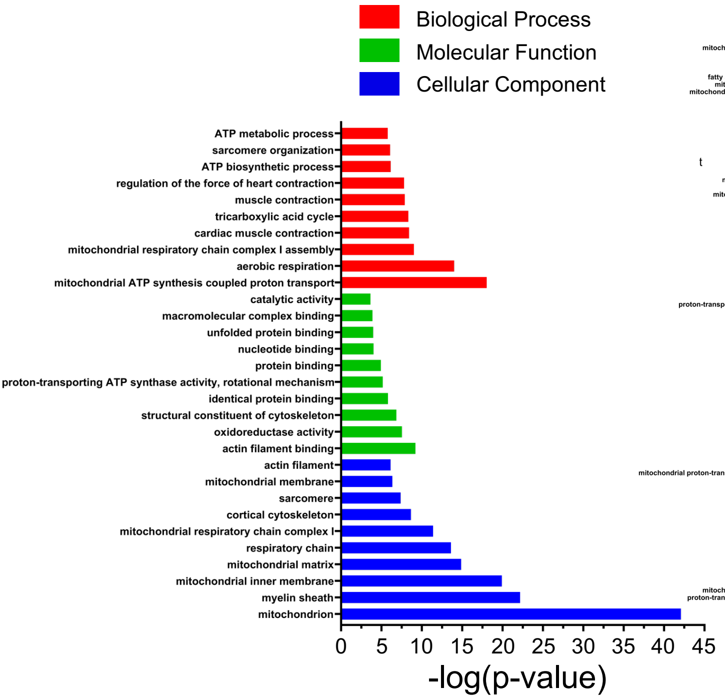

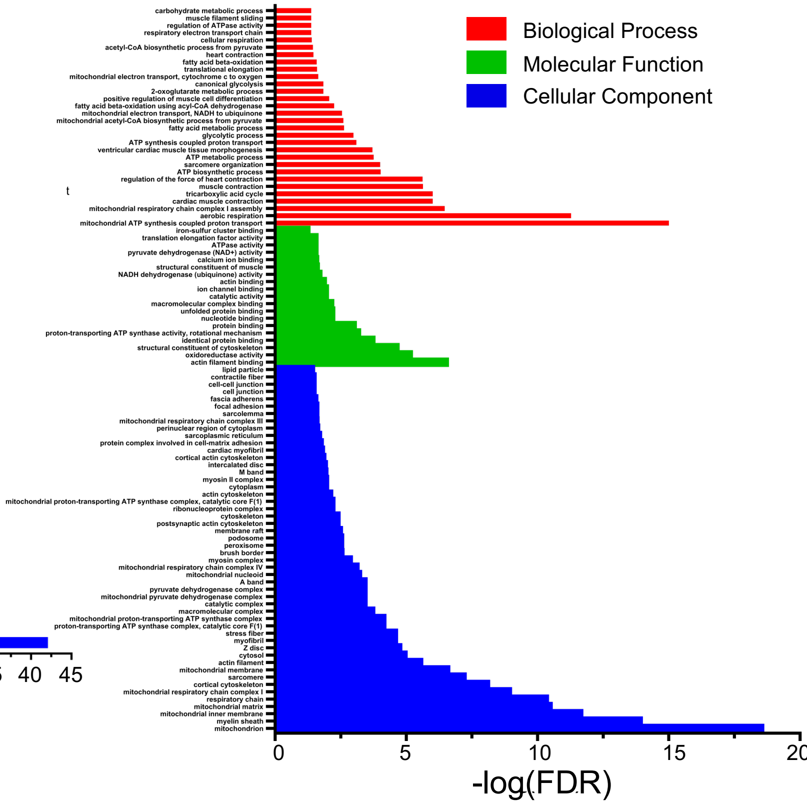

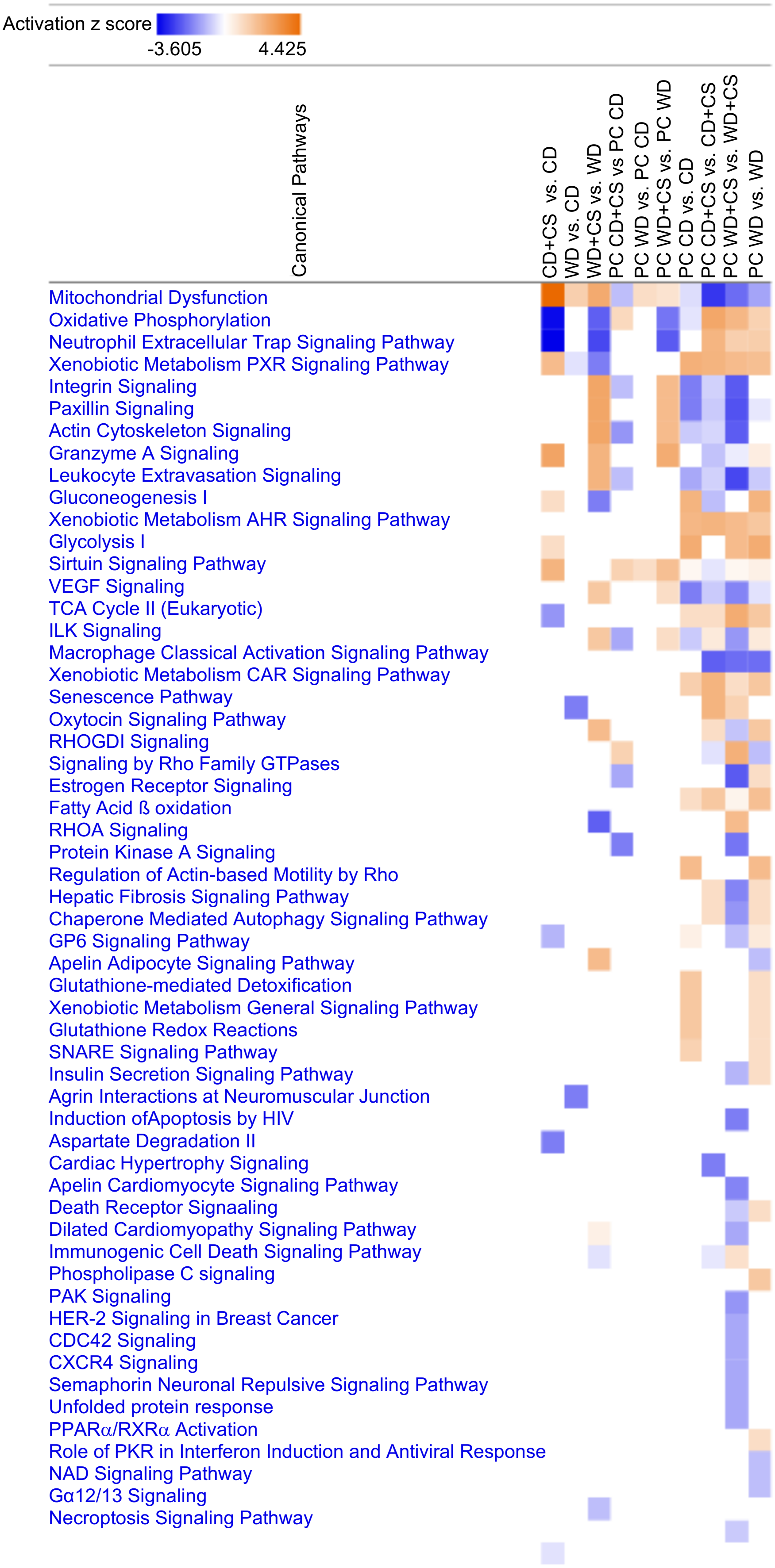

Fig. S10. Patterns of canonical pathway changes in post-ischemic hearts from CD, WD, CD+CS and WD+CS mice (± adaptive PC)

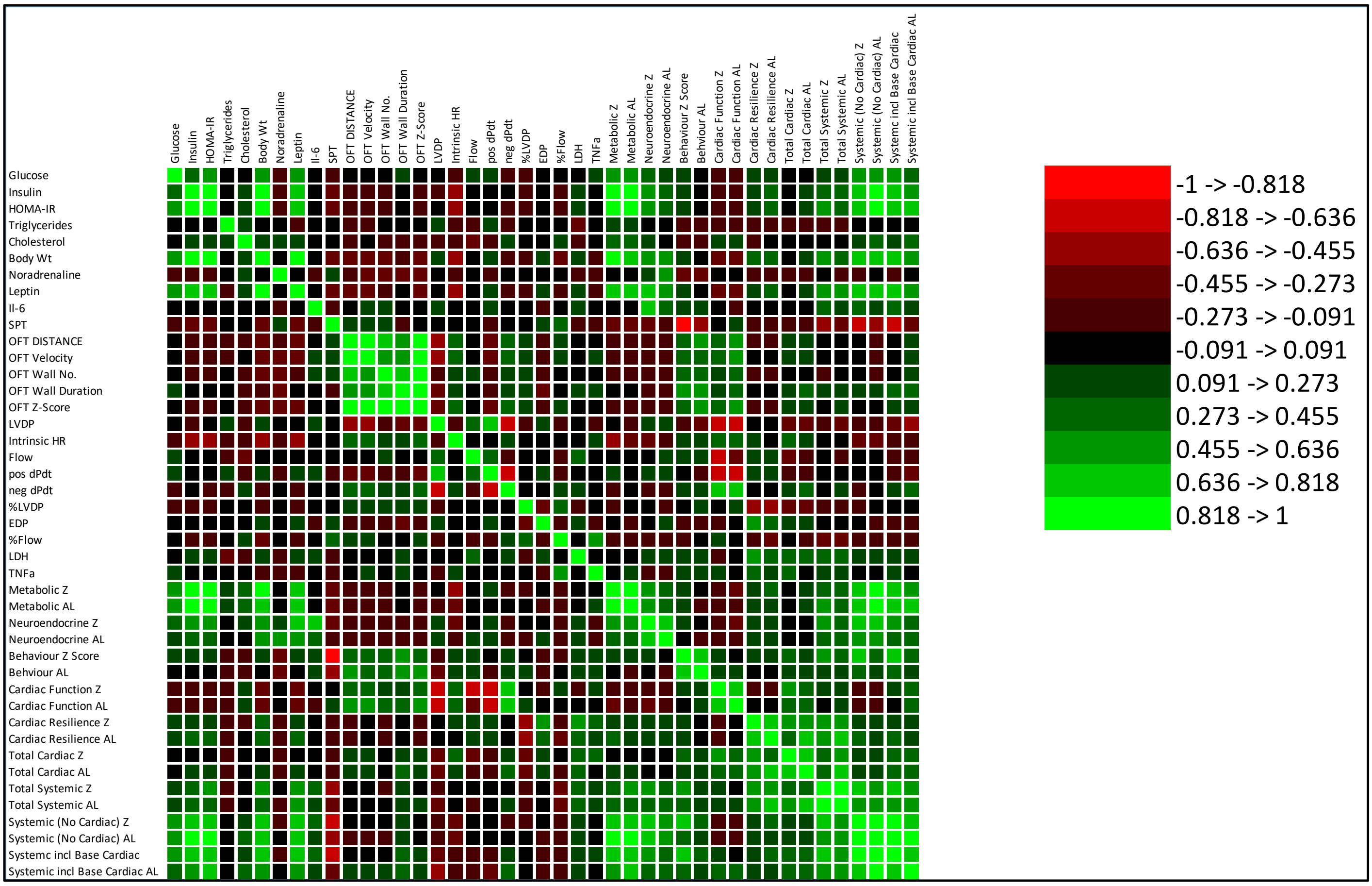

Fig. S11. Regression matrix (Pearson coefficient from linear regression) for variables from CD, WD, CD+CS and WD+CS mice and hearts.
